# Supplementary material for: Outcomes following early parenteral nutrition use in preterm neonates: protocol for an observational study
Source: BMJ Open. 2019 Jul 9;9(7):e029065. doi: 10.1136/bmjopen-2019-029065 (PMC6615825; doi:10.1136/bmjopen-2019-029065)
Supplement: Supplementary data [file bmjopen-2019-029065supp001.pdf]

Online only supplemental material

eTable 1: Exclusion criteria for comparative study: list of major congenital gastrointestinal malformations
eTable 2: Exclusion criteria for comparative study: list of life-limiting conditions or conditions requiring surgery in the neonatal period
eText 1: Data fields for extraction from NNRD

eTable 1: Exclusion criteria for comparative study: list of major congenital gastrointestinal malformations

Cleverme d code	ICD-10 code	Diagnosis
10741	Q39.0	Oesophageal atresia without distal fistula
16195	Q39.0	Atresia of oesophagus without fistula
10740	Q39.1	Oesophageal atresia with distal tracheo-oesophageal fistula
16196	Q39.1	Atresia of oesophagus with tracheo-oesophageal fistula (TOF)
16197	Q39.2	Congenital tracheo-oesophageal fistula without atresia (TOF)
10273	Q39.3	Congenital stenosis of the oesophagus
16198	Q39.3	Congenital stenosis and stricture of oesophagus
16199	Q39.4	Oesophageal web
10358	Q41.0	Duodenal atresia / stenosis / web (specify)
16212	Q41.0	Congenital absence, atresia and stenosis of duodenum
16213	Q41.0DA	Duodenal atresia / stenosis
10605	Q41.1	Jejunal atresia / stenosis (specify)
16214	Q41.1JA	Jejunal atresia / stenosis
10541	Q41.2	Ileal atresia / stenosis (specify)
16215	Q41.2	Congenital absence, atresia and stenosis of ileum
16216	Q41.2IA	Ileal atresia / stenosis
16217	Q41.X	Congenital absence, atresia and stenosis of small intestine
16218	Q42.0	Congenital absence, atresia and stenosis of rectum with fistula
10496	Q42.00	High anorectal anomaly with rectourethral fistula
10497	Q42.01	High anorectal anomaly with rectovesical fistula
10498	Q42.02	High anorectal anomaly with rectovulval fistula
10495	Q42.03	High anorectal anomaly with rectocutaneous fistula
10494	Q42.04	High anorectal anomaly with rectocloacal fistula
10493	Q42.08	High anorectal anomaly with fistula (specify)
10499	Q42.1	High anorectal anomaly without fistula
16219	Q42.1	Congenital absence, atresia and stenosis of rectum without fistula
16220	Q42.2	Congenital absence, atresia and stenosis of anus with fistula
10636	Q42.20	Low anorectal anomaly with anocutaneous fistula
10637	Q42.21	Low anorectal anomaly with anovestibular fistula
10638	Q42.28	Low anorectal anomaly with fistula (other specify)
10639	Q42.3	Low anorectal anomaly without fistula
16221	Q42.3	Congenital absence, atresia and stenosis of anus without fistula
10240	Q42.31	Congenital anal stenosis
16222	Q42.8	Congenital absence, atresia and stenosis of anus of other parts of large intestine
16223	Q429	Congenital absence, atresia and stenosis of anus of large intestine, part unspecified
16224	Q42X	Congenital absence, atresia and stenosis of large intestine

16235	Q43.7	Persistent cloaca

eTable 2: Exclusion criteria for comparative study: list of life-limiting conditions or conditions requiring surgery in the neonatal period

Clevermed code	ICD-10 code	Diagnosis
15890	Q00.0	Anencephaly
15891	Q00.1	Craniorachischisis
15892	Q00.2	Iniencephaly
15893	Q00.X	Anencephaly and similar malformations
15894	Q01.0	Frontal encephalocele
15895	Q01.1	Nasofrontal encephalocele
15896	Q01.2	Occipital encephalocele
15897	Q01.8	Encephalocele of other sites
15898	Q01.9	Encephalocele (unknown or unspecified cause)
15899	Q01.X	Encephalocele
15918	Q04.2	Holoprosencephaly
15926	Q05.0	Cervical spina bifida with hydrocephalus
15927	Q05.1	Thoracic spina bifida with hydrocephalus
15928	Q05.2	Lumbar spina bifida with hydrocephalus
15929	Q05.3	Sacral spina bifida with hydrocephalus
15930	Q05.4	(unknown or unspecified cause) spina bifida with hydrocephalus
15931	Q05.5	Cervical spina bifida without hydrocephalus
15932	Q05.6	Thoracic spina bifida without hydrocephalus
15933	Q05.7	Lumbar spina bifida without hydrocephalus
15934	Q05.8	Sacral spina bifida without hydrocephalus
15935	Q05.9	Spina bifida (unknown or unspecified cause)
10986	Q05.9a	Spina bifida
10704	Q05.9b	Myelomeningocele (specify site)
15936	Q05.X	Spina bifida
16024	Q20.0	Common arterial trunk (Truncus malformation)
10356	Q20.1	Double outlet right ventricle (DORV)
16025	Q20.1	Double outlet right ventricle (DORV)
16026	Q20.2	Double outlet left ventricle (DOLV)
11070	Q20.3	Transposition of the great vessels (TGA)
16027	Q20.3	Transposition great arteries (TGA)
16028	Q20.4	Double inlet ventricle (DILV)
16029	Q20.5	Discordant atrioventricular connection
16030	Q20.6	Isomerism of atrial appendages
16031	Q20.8	Other cong malforms of cardiac chambers and connections
16032	Q20.9	Cong malforms of cardiac chambers and connections unspec
16033	Q20.X	Congenital malformations of cardiac chambers and connections
16035	Q20.91	Atrium single

16036	Q20.92	Ventricle single
10097	Q21.2	Atrio-ventricular septal defect (AVSD)
16039	Q21.2	Atrioventricular septal defect (AVSD)
11043	Q21.3	Tetralogy of Fallot
16040	Q21.3	Tetralogy of Fallot
16045	Q22.0	Pulmonary valve atresia
16046	Q22.1	Congenital pulmonary valve stenosis
16047	Q22.2	Congenital pulmonary valve insufficiency
16048	Q22.3	Other congenital malformations of pulmonary valve
16049	Q22.4	Congenital tricuspid atresia / stenosis
16050	Q22.5	Ebstein's anomaly
16051	Q22.6	Hypoplastic right heart syndrome
16052	Q22.8	Other congenital malformations of tricuspid valve
16053	Q22.9	Congenital malformation of tricuspid valve (unknown or unspecified cause)
16054	Q22.X	Congenital malformations of pulmonary and tricuspid valves
16055	Q23.0	Congenital stenosis of aortic valve (AS)
16056	Q23.1	Congenital insufficiency of aortic valve
16057	Q23.2	Congenital mitral stenosis (MS)
16058	Q23.3	Mitral atresia
16059	Q23.4	Hypoplastic left heart syndrome (HLH)
16060	Q23.8	Other congenital malformations of aortic and mitral valves
16061	Q23.9	Congenital malformation of aortic and mitral valves unspec
16062	Q23.X	Congenital malformations of aortic and mitral valves
16079	Q25.1	Coarctation of aorta
10227	Q25.19	Coarctation of the aorta
16080	Q25.2	Hypoplasia of aortic arch
16081	Q25.3	Stenosis of aorta (AS)
16082	Q25.4	Malformation of aorta
16083	Q25.5	Atresia of pulmonary artery
16084	Q25.6	Stenosis of pulmonary artery (PS)
16086	Q25.8	Other congenital malformations of great arteries
16087	Q25.8	Transposition of the great vessels (TGA)
11057	Q26.2	Total anomylous pulmonary venous drainage (TAPVD)
16092	Q26.2	Total anomalous pulmonary venous connection (TAPVD)
16154	Q33.6	Hypoplasia and dysplasia of lung
16241	Q44.2	Atresia of bile ducts
10123	Q60.1	Bilateral renal agenesis
16318	Q60.1B	Renal agenesis, bilateral
16324	Q60.6	Potter's syndrome
16327	Q61.1	Polycystic kidney, infantile type
10100	Q61.1a	Autosomal recessive polycystic kidney - infantile

10367	Q64.1	Ectopia vesicae
16356	Q64.1	Exstrophy of urinary bladder
10854	Q64.2	Posterior urethral valves (PUV)
16357	Q64.2	Congenital posterior urethral valves (PUV)
16360	Q64.5	Congenital absence of bladder and urethra
10008	Q64.5a	Absence of bladder
10236	Q64.5b	Congenital absence of urethra
16475	Q77.1	Thanatophoric short stature
10246	Q79.0	Congenital diaphragmatic hernia
10490	Q79.0	Hernia into the cord
16495	Q79.0	Congenital diaphragmatic hernia
16496	Q79.1A	Aplasia of diaphragm
16497	Q79.1E	Eventration of diaphragm
16498	Q79.2	Exomphalos
10395	Q79.2	Exomphalos
16499	Q79.3	Gastroschisis
16589	Q90.0	Trisomy 21, meiotic nondisjunction
16590	Q90.1	Trisomy 21, mosaicism (mitotic nondisjunction)
16591	Q90.2	Trisomy 21, translocation
16592	Q90.9	Down's syndrome (unknown or unspecified cause)
16593	Q90.X	Down's syndrome
16594	Q91.0	Trisomy 18, meiotic nondisjunction
16595	Q91.1	Trisomy 18, mosaicism (mitotic nondisjunction)
16596	Q91.2	Trisomy 18, translocation
16597	Q91.3	Edwards' syndrome (unknown or unspecified cause)
16598	Q91.4	Trisomy 13, meiotic nondisjunction
16599	Q91.5	Trisomy 13, mosaicism (mitotic nondisjunction)
16600	Q91.6	Trisomy 13, translocation
16601	Q91.7	Patau's syndrome (unknown or unspecified cause)
16602	Q91.X	Edwards' syndrome and Patau's syndrome

eText 1: Data fields for extraction from NNRD

**Treatment**	
**Variable**	**Data items**
Parenteral nutrition	**PN group** defined as<br>Any of the following items entered in the 'Daily Care Fluids' and 'Feeding' during first 7 days:<br>• Y entry for PARENTERAL NUTRITION RECEIVED INDICATOR<br>Or<br>The following drug code entered in the Daily care medication during first 7 days:<br>• 1010238 Total parenteral nutrition<br><br>**No PN group** defined as<br>All other babies

**Background variables for matching**	
**Variable**	**Data items**
Gestational age at birth	**30^{+0} to 30^{+6} group** defined as<br>Any of the following items entered in the GESTATION LENGTH (AT DELIVERY):<br>• 30^{+0}, 30^{+1}, 30^{+2}, 30^{+3}, 30^{+4}, 30^{+5}, 30^{+6}<br><br>**31^{+0} to 31^{+6} group** defined as<br>Any of the following items entered in the GESTATION LENGTH (AT DELIVERY):<br>• 31^{+0}, 31^{+1}, 31^{+2}, 31^{+3}, 31^{+4}, 31^{+5}, 31^{+6}<br><br>**32^{+0} to 32^{+6} group** defined as<br>Any of the following items entered in the GESTATION LENGTH (AT DELIVERY):<br>• 32^{+0}, 32^{+1}, 32^{+2}, 32^{+3}, 32^{+4}, 32^{+5}, 32^{+6}
Small for gestational age	**Small for gestational age group** defined as<br>Any result entered in the BIRTH WEIGHT which is below the 10th centile on the UK-WHO growth chart<br><br>**Appropriate for gestational age group** defined as<br>All other babies

**Background variables for propensity score matching**	
**Variable**	**Data items**
Sex	Data will be extracted from PERSON PHENOTYPIC SEX<br>• Categorical: 1 Male / 2 Female / 9 Indeterminate (unable to be classified as either male or female)
Multiplicity	Data will be extracted from NUMBER OF FETUSES (NOTED DURING PREGNANCY EPISODE); this excludes fetus papyraceous and fetuses reabsorbed in utero and not delivered.
Year of birth	Data will be extracted from DATE TIME OF BIRTH<br>• Continuous in one year bands
Maternal age	Data will be extracted from YEAR OF BIRTH (MOTHER)<br>• Continuous variable
Maternal diabetes	Data will be extracted from MATERNITY COMPLICATING MEDICAL DIAGNOSIS TYPE (NATIONAL NEONATAL DATA SET)<br>• Dichotomous: 08 Y/N
Maternal gestational	Data will be extracted from MATERNITY OBSTETRIC DIAGNOSIS

diabetes	TYPE (CURRENT PREGNANCY)<br>• Dichotomous: 06 Gestational diabetes mellitus Y/N
Maternal severe pre-eclampsia requiring pre-term birth	Data will be extracted from MATERNITY OBSTETRIC DIAGNOSIS TYPE (CURRENT PREGNANCY)<br>• Dichotomous: 01 Severe pre-eclampsia requiring pre-term birth Y/N
Maternal severe pre-eclampsia	Data will be extracted from MATERNITY OBSTETRIC DIAGNOSIS TYPE (CURRENT PREGNANCY)<br>• Dichotomous: 20 Severe pre-eclampsia Y/N
Maternal gestational hypertension	Data will be extracted from MATERNITY OBSTETRIC DIAGNOSIS TYPE (CURRENT PREGNANCY)<br>• Dichotomous: 07 Gestational hypertension Y/N
Maternal prolonged rupture of membranes	Data will be extracted from NUMBER OF MINUTES (BIRTH TO EVENT)<br>• Continuous variable
Maternal suspected chorioamnionitis	Data will be extracted from SIGNIFICANT MATERNAL PYREXIA IN LABOUR INDICATOR or INTRAPARTUM ANTIBIOTICS GIVEN INDICATOR<br>• Dichotomous: Suspected chorioamnionitis defined as Y in either field<br>• Dichotomous: No suspected chorioamnionitis defined as N in both fields
Maternal receipt of antenatal steroids	Data will be extracted from STEROIDS GIVEN DURING PREGNANCY TO MATURE FETAL LUNGS INDICATOR (Y/N) and ANTENATAL STEROID COURSE COMPLETION STATUS<br>• Categorical: Complete course defined as Y and 1 Complete course<br>• Categorical: Incomplete course defined as Y and 2 Incomplete course<br>• Categorical: No steroids defined as N
Maternal receipt of antenatal magnesium sulphate	Data will be extracted from MOTHER RECEIVED MAGNESIUM SULPHATE IN 24 HOURS PRIOR TO DELIVERY<br>• Dichotomous: Y/N
Infant Apgar score at 5 minutes	Data will be extracted from APGAR SCORE (5 MINUTES)<br>• Categorical: 0-10
Infant: chest compressions administered	Data will be extracted from NEONATAL RESUSCITATION METHODS (NATIONAL NEONATAL DATA SET)<br>• Dichotomous: 16 Cardiac massage (Y/N)
Infant: Emergency resuscitation drugs administered	Data will be extracted from NEONATAL RESUSCITATION METHODS (NATIONAL NEONATAL DATA SET)<br>• Dichotomous: 17 Adrenaline or 88 Any other drug (Y/N)
Infant: Intubated at resuscitation	Data will be extracted from NEONATAL RESUSCITATION METHODS (NATIONAL NEONATAL DATA SET)<br>• Dichotomous: 15 Intubation (Y/N)
Infant: Surfactant administered	Data will be extracted from SURFACTANT GIVEN INDICATOR (DURING RESUSCITATION)<br>• Dichotomous: Y/N
Infant: Umbilical cord pH	Data will be extracted from UMBILICAL CORD BLOOD PH LEVEL (ARTERIAL)<br>• Continuous: Arterial cord pH (6.00-8.00)<br>Or if unavailable use:<br>UMBILICAL CORD BLOOD PH LEVEL (VENOUS)<br>• Continuous: Venous cord pH (6.00-8.00)
Infant: Admission temperature	Data will be extracted from TEMPERATURE (ON ADMISSION TO NEONATAL CRITICAL CARE)<br>• Continuous
Infant: Admission mean blood pressure	Data will be extracted from MEAN ARTERIAL BLOOD PRESSURE (ON ADMISSION TO NEONATAL CRITICAL CARE)

	• Continuous: 10-150
Infant: Admission blood glucose	Data will be extracted from BLOOD GLUCOSE CONCENTRATION (ON ADMISSION TO NEONATAL CRITICAL CARE)<br>• Continuous: 0.0-50.0
Infant: Admission heart rate	Data extracted from HEART RATE (ON ADMISSION TO NEONATAL CRITICAL CARE)<br>• Continuous: 50-350
Infant: Admission respiratory rate	Data extracted from RESPIRATORY RATE (ON ADMISSION TO NEONATAL CRITICAL CARE)<br>• Continuous: 10-200
Infant: Admission oxygen saturation	Data extracted from OXYGEN SATURATION (ON ADMISSION TO NEONATAL CRITICAL CARE)<br>• Continuous: 10-100
Infant: Surfactant administered on first day	Data extracted from SURFACTANT GIVEN INDICATOR (ON NEONATAL CRITICAL CARE DAILY CARE DATE)<br>• Continuous: Y/N
Infant: Mechanical ventilation on first day	Data extracted from RESPIRATORY SUPPORT DEVICE TYPE (NATIONAL NEONATAL DATA SET) for first day<br>• Mechanical ventilation defined as 1 Endotracheal tube<br>• No ventilation defined as any other answer
Infant: Inotropes administered on first day	Data extracted from INOTROPE INFUSION RECEIVED INDICATOR for first day<br>• Dichotomous: Y/N<br>Or DAILY CARE MEDICATION on day 1 only<br>• 500098 Dopamine<br>• 500096 Dobutamine<br>• 500056 Adrenaline<br>• 500210 Noradrenaline<br>• 500116 Hydrocortisone<br>• 1010173 Milrinone
Infant: Sepsis suspected on first day	Data extracted from DAILY CARE INFECTIONS SEPSIS SUSPECTED INDICATOR for first day<br>• Y/N
Infant: Transfer on first day	Data extracted from Admission Details SITE CODE (OF ADMITTING NEONATAL UNIT) or ORGANISATION CODE (OF ADMITTING NEONATAL UNIT) is different from Baby Demographics SITE CODE (OF ACTUAL PLACE OF DELIVERY) or ORGANISATION CODE (OF ACTUAL PLACE OF DELIVERY)<br>And Baby Demographics EPISODE NUMBER is >1
Level of initial neonatal unit	Data extracted from SITE CODE (OF ACTUAL PLACE OF DELIVERY)
Neonatal network	Data extracted from SITE CODE (OF ACTUAL PLACE OF DELIVERY)
Enteral feeding	Data extracted from DAILY CARE FLUIDS AND FEEDING ENTERAL FEED TYPE GIVEN on day 1 and 2<br>• Categorical: Only maternal milk feeding defined as any of 1 Breastfeeding, 2 Mothers fresh expressed breast milk, 3 Mothers frozen expressed breast milk on either day with no other code.<br>• Categorical: Only donor milk feeding defined as 4 Donor expressed breast milk on either day with no other code.<br>• Categorical: Only formula defined as only 6 Formula milk on either day with no other code.<br>• Categorical: Not feeding defined as 9 - Not applicable (nil by mouth) on both days with no other code.<br>• Categorical: Mixed feeding as any combination of codes not consistent with the above categories.

Outcomes	
**Variable**	**Data items**
Survival	Data extracted from DISCHARGE DESTINATION FROM NEONATAL CRITICAL CARE<br>• Survival defined as any of 1, 2, 4, 5, 6<br>• Died defined as code 3, Died
Late onset sepsis	NNAP definition<br>Defined from Infection Cultures (Episodic) recorded after day 3<br>• Pure growth of pathogen from blood OR Pure growth of pathogen from CSF OR Either a pure growth of a skin commensal or a mixed growth with ≥3 clinical signs at the time of blood sampling
Necrotising enterocolitis	NNAP definition<br>Defined from DISCHARGE DETAILS based on WAS NEC DIAGNOSED THIS ADMISSION answer Y<br>With at least one clinical feature from:<br>• Bilious gastric aspirate or emesis/Abdominal distension/Occult or gross blood in stool (no fissure)<br>And at least one radiographic feature from:<br>• Pneumatosis/Hepato-biliary gas/Pneumoperitoneum<br><br>Where NNAP definition not recorded (e.g. prior to 2016):<br>Defined from Daily Care Gastrointestinal at any point during neonatal unit stay:<br>• Any entry (1 or 2) for TREATMENT TYPE FOR NECROTISING ENTEROCOLITIS<br>Or: From diagnostic codes •<br>• 1010683 Necrotising enterocolitis – suspected<br>• 10708 Necrotising enterocolitis – Perforated<br>• 15809 Necrotizing enterocolitis<br><br>AND<br><br>5 or more days nil by mouth defined from the Daily Care Fluids and Feeding for a continuous period of 5 days<br>• No under ENTERAL FEED TYPE GIVEN<br>• No entry under FORMULA MILK OR MILK FORTIFIER TYPE<br>• No value OR 0 for TOTAL VOLUME OF MILK RECEIVED<br>• No entry under ENTERAL FEEDING METHOD<br>While also receiving 5 or more days of antibiotics over the same 5 days as the baby was nil by mouth<br>Defined as 5 consecutive days of any of the following Daily care medication<br>• 1010155 Benzyl Penicillin<br>• 1010158 Augmentin<br>• 1010179 Flucloxicillin<br>• 500012 Flucloxacillin<br>• 500016 Gentamicin<br>• 500072 Co-amoxiclav<br>• 500086 Co-amoxiclav<br>• 500084 Ciprofloxacin<br>• 500029 Netilmicin<br>• 500002 Amikacin<br>• 500211 Tazocin<br>• 500023 Metronidazole<br>• 500040 Vancomycin<br>• 500007 Cefotaxime<br>• 500004 Ampicillin

	• 500009 Cefuroxime<br>• 500008 Ceftazidime<br>• 500175 Ceftriaxone<br>• 500032 Piperacillin<br>• 500206 Oflacillin<br>• 500005 Azlocillin<br>• 1010171 Linezolid<br>• 1010271 Cefalexin<br>• 1010139 Amoxicillin<br>• 500070 Amoxicillin<br>• 500128 Meropenem<br>• 500118 Imepenem<br>• 500145 Imipenem
Brain injury on imaging	Data extracted from CRANIAL ULTRASOUND SCANS (EPISODIC)<br>Brain injury defined as:<br>• INTRAVENTRICULAR HAEMORRHAGE GRADE (RIGHT SIDE) or INTRAVENTRICULAR HAEMORRHAGE GRADE (LEFT SIDE) code 3 or 4 (Grade 3/4 intraventricular haemorrhage)<br>Or:<br>• CYSTIC PERIVENTRICULAR LEUKOMALACIA OBSERVED DURING CRANIAL ULTRASOUND SCAN INDICATOR answer Y
Retinopathy of prematurity	Data extracted from RETINOPATHY OF PREMATURITY SCREENING (EPISODIC)<br>Retinopathy of prematurity defined as:<br>• RETINOPATHY OF PREMATURITY STAGE (LEFT EYE) or RETINOPATHY OF PREMATURITY STAGE (RIGHT EYE) any code except 0 (None seen)
Bronchopulmonary dysplasia	NNAP definition<br>Significant bronchopulmonary dysplasia defined as:<br>• DAILY SUMMARY at 36^{+0} receiving any respiratory support
Need for surgical procedures	Data extracted from PROCEDURE (OPCS ON NEONATAL CRITICAL CARE DAILY CARE DATE)<br>Surgery defined as any of the following codes:<br>• 100033 Surgery for meconium ileus (von)<br>• 100076 Skin or soft tissue surgery requiring general or spinal anesthesia (Description Required<br>• 11222 Closure of small intestine/ileal perforation<br>• 11501 Laparoscopy<br>• 11904 Colostomy<br>• 11905 Ileostomy<br>• 1010826 Major surgery<br><br>Or:<br>• Daily item ANY MAJOR SURGERY TODAY answer Y
Seizures	Seizure defined as:SEIZURE OCCURRED INDICATOR<br>• Y<br>Or: DIAGNOSIS (ICD RECORDED ON DISCHARGE FROM NEONATAL CRITICAL CARE) with code<br>• 10957 Seizures<br>• 15192 Seizure disorder<br>• 15194 Seizure disorder (cause unknown)<br>• 15195 Status epilepticus<br>• 15848 Seizures
Weight	Data extracted from Daily Care General Information PERSON WEIGHT IN GRAMS from final day
Head circumference	Data extracted from Daily Care General Information HEAD

	CIRCUMFERENCE IN CENTIMETRES from final day

**Long Term Outcomes**	
**Variable**	**Data items**
Blindness	Defined as an answer of Yes to the question “Does this child have a visual impairment?” on the NNAP form
Deafness	Defined as an answer of Yes to the question “Does this child have a hearing impairment?” on the NNAP form
Ability to walk	Defined as an answer of Yes to the question “Is this child unable to walk without assistance?” on the NNAP form
