# Supplementary material for: Outcomes following early parenteral nutrition use in preterm neonates: protocol for an observational study
Source: BMJ Open. 2019 Jul 9;9(7):e029065. doi: 10.1136/bmjopen-2019-029065 (PMC6615825; doi:10.1136/bmjopen-2019-029065)
Supplement: Author's manuscript [file bmjopen-2019-029065.draft_revisions.pdf]

BMJ Open

BMJ Open is committed to open peer review. As part of this commitment we make the peer review history of every article we publish publicly available.

When an article is published we post the peer reviewers' comments and the authors' responses online. We also post the versions of the paper that were used during peer review. These are the versions that the peer review comments apply to.

The versions of the paper that follow are the versions that were submitted during the peer review process. They are not the versions of record or the final published versions. They should not be cited or distributed as the published version of this manuscript.

BMJ Open is an open access journal and the full, final, typeset and author-corrected version of record of the manuscript is available on our site with no access controls, subscription charges or pay-per-view fees (http://bmjopen.bmj.com).

If you have any questions on BMJ Open's open peer review process please email info.bmjopen@bmj.com

BMJ Open

Outcomes following early parenteral nutrition use in preterm neonates: Protocol for an observational study

Journal:	*BMJ Open*
Manuscript ID	bmjopen-2019-029065
Article Type:	Protocol
Date Submitted by the Author:	10-Jan-2019

Complete List of Authors:	Webbe, James; Imperial College London, Section of Neonatal Medicine<br>Longford, Nick; Imperial College London, Section of Neonatal Medicine<br>Uthaya, Sabita; Imperial College London,<br>Modi, Neena; Imperial College London, Medicine<br>Gale, Chris; Imperial College London, Section of Neonatal Medicine;

Keywords:	NEONATOLOGY, Neonatal intensive & critical care < INTENSIVE & CRITICAL CARE, Nutritional support < GASTROENTEROLOGY

SCHOLARONE™
Manuscripts

Outcomes following early parenteral nutrition use in preterm neonates: Protocol for an observational study

James Webbe[1], Nicholas Longford[2], Sabita Uthaya[3], Neena Modi[4], Chris Gale[5]

[1] James Webbe

Section of Neonatal Medicine, Imperial College London

London, United Kingdom.

[2] Nicholas Longford

Section of Neonatal Medicine, Imperial College London

London, United Kingdom.

[3] Sabita Uthaya

Section of Neonatal Medicine, Imperial College London

London, United Kingdom.

[4] Neena Modi

Section of Neonatal Medicine, Imperial College London

London, United Kingdom.

[5] Chris Gale, Clinical Senior Lecturer

Section of Neonatal Medicine, Chelsea and Westminster campus, Imperial College London

369 Fulham Road, London, SW10 9NX, United Kingdom.

Corresponding author:

Dr James Webbe, Clinical Research Fellow

For peer review only

2

Section of Neonatal Medicine, Chelsea and Westminster Campus, Imperial College London

369 Fulham Road, London, SW10 9NX, United Kingdom.

+ 44 (0)203 3155418

james.webbe@imperial.ac.uk

Manuscript word count: 2655

For peer review only

Abstract

Introduction

Preterm babies are among the highest users of parenteral nutrition (PN) of any patient group, but there is wide variation in commencement, duration, and composition of PN and uncertainty around which groups will benefit from early introduction. Recent studies in critically unwell adults and children suggest that harms, specifically increased rates of nosocomial infection, outweigh the benefits of early administration of PN. In this study we will describe early PN use in neonatal units in England, Wales and Scotland. We will also evaluate if this affects survival and other important neonatal outcomes in neonates born between 30^{+0} and 32^{+6} $weeks^{+days}$ gestation.

Methods and analysis

We will use routinely collected data from all neonatal units in England, Wales and Scotland, available in the National Neonatal Research Database (NNRD) at Imperial College London. We will describe clinical practice in relation to any use of PN during the first seven postnatal days among neonates admitted to neonatal care between 1st January 2012 and 31st December 2017. We will compare outcomes in neonates born between 30^{+0} and 32^{+6} $weeks^{+days}$ gestation who did or did not receive PN in the first week after birth using a propensity score matched approach. The primary outcome will be survival to discharge home. Secondary outcomes will include components of the neonatal core outcome set; outcomes identified as important by former patients, parents, clinicians and researchers.

Regulatory approvals and dissemination

We have obtained UK National Research Ethics Committee approval for this study (Ref: 18/NI/0214). The results of this study will be presented at academic conferences; the United Kingdom charity Bliss will aid dissemination to former patients and parents.

For peer review only

4

Registration

ClinicalTrials.gov: NCT03767634

Registration date: 06/12/2018

Keywords

[1] Parenteral nutrition [2] Preterm neonate [3] Core outcomes set [4] Propensity score matching [5] Neonatal care [6] National Neonatal Research database

Abstract word count: 298

For peer review only

ARTICLE SUMMARY

Strengths and limitations of this study

- We will use routinely recorded data held in the National Neonatal Research Database (NNRD); these have been shown to be complete and of high quality
- The NNRD covers the entire neonatal unit population of England, Wales and Scotland and thus provides power to explore how early PN use affects outcomes
- As a retrospective, observational study results will be vulnerable to confounding
- The NNRD contains a large number of variables that will assist in propensity score matching to form well-balanced groups, diminishing potential confounding
- We will focus on core outcomes identified as important by former patients and parents as well as clinicians and researchers

For peer review only

Introduction

Preterm birth abruptly ends the transplacental transfer of nutrients essential for fetal growth and development. Providing adequate nutrition is an essential component of neonatal care but very preterm infants often have difficulty in tolerating adequate volumes of milk immediately after birth (1). To meet their nutritional needs such infants are commonly given supplemental parenteral nutrition (PN). Preterm babies are among the highest PN users of all NHS patients but clinical practice is variable both nationally and internationally: some neonatal units in high-income countries are reported to provide PN to around 70% of neonatal admissions (2), while others report using no PN (3). In the United Kingdom national guidelines recommend administration of PN to all babies born below 30 weeks gestational age (4), however exact rates of PN use in UK neonatal units are not known. When considering the efficacy of PN use in neonates, although the impact of early PN on nitrogen balance is known (5), the effects on survival, growth and neurodevelopment are less clear (5, 6). Despite widespread use, the impact of administration of PN on key outcomes has not been evaluated in randomised, controlled neonatal trials powered for clinical end-points.

Parenteral nutrition carries well established risks, of which the most serious and common is blood stream infection (7). There is a growing body of evidence that use of PN in critically unwell adults (8) and children (9) within the first seven days of admission to an intensive care unit is associated with worse outcomes. Furthermore, a subgroup analysis of a paediatric intensive care unit population limited to term neonates less than 28 days old showed an increase in nosocomial infection with early PN use (10). These studies raise uncertainty over the balance of risks and benefits of PN administration in the early postnatal period. It is generally accepted that PN is likely to be beneficial in extremely preterm neonates where postnatal nutrient deficits are most severe and prolonged, but in moderately preterm neonates the effect of PN use on neonatal survival or other key outcomes has never

For peer review only

been conclusively demonstrated (5, 11, 12). The largest randomised trials to date (13-15) have recruited fewer than 100 babies to each interventional arm and were therefore only powered to detect very large (10%) absolute risk differences in mortality or other outcomes. Neonates are vulnerable to unanticipated treatment effects across different organ systems (16), therefore it is important to show that PN does not adversely impact any important neonatal outcomes in addition to the nutrition outcomes commonly studied in PN trials. The scant evidence-base to inform use is reflected in variation in timing of commencement, duration of use and composition of PN within and between countries (17, 18).

This protocol describes how we will approach the following research questions:

- What is the pattern of PN use in neonatal units in England, Scotland and Wales in the first seven postnatal days?
- In neonates born between 30^{+0} and 32^{+6} weeks gestational age, is PN use in the first seven postnatal days, compared with no use of PN, associated with altered survival to discharge home?
- For neonates born between 30^{+0} and 32^{+6} weeks gestational age is use of PN in the first seven postnatal days, compared with no use of PN, associated with different core neonatal outcomes?

Methods

Study design

This study contains two projects that will use data held in the National Neonatal Research Database. First we will evaluate practice, and then we will undertake a comparison of matched groups of moderately preterm babies.

Data source

For peer review only

This study will use de-identified data held in the NNRD (19). The NNRD holds data extracted from point-of-care electronic health records completed by health professionals during routine clinical care (20). The Neonatal Data Set, a defined national data standard (21) comprising approximately 450 items, is extracted and transmitted to the Neonatal Data Analysis Unit at Imperial College London. The data set includes demographic items relating to mother and baby (e.g. gestational age at birth, birthweight, maternal conditions), daily items (e.g. feeding information, administration of PN), ad hoc items (e.g. suspected infection, cranial ultrasound findings), and discharge items (e.g. diagnoses during admission, weight at discharge). It contains data about which hospital a baby is born in and which neonatal network (the managed clinical networks that provide neonatal care within a geographical are) each hospital is part of (22). The NNRD holds data from all infants admitted to NHS neonatal units in England, Scotland and Wales (approximately 90,000 infants each year). In total the NNRD contains data from approximately one million infants from 2008 to the present. All units in England and Wales have contributed data since 2012 and all Scottish units since 2015. The completeness and quality of data held in the NNRD has been shown to be high (23), making it suitable for research (24).

For peer review only

Eligibility

For the description of practice we will utilise data on all neonates born between 1st January 2012 and 31st December 2017 and admitted to a neonatal unit in England, Scotland and Wales without.

For the matched comparative study we will use data on all neonates born between 30^{+0} and 32^{+6} weeks gestational age between 1st January 2012 and 31st December 2017 and admitted to a neonatal unit in England, Scotland and Wales. Neonates with major congenital gastrointestinal malformations will be excluded as they cannot be fed enterally (eTable 1). Neonates with life limiting conditions (25) or congenital conditions requiring surgery in the neonatal period will be excluded as they will not receive standard neonatal

nutritional care (eTable 2). Both groups would bias results because they will fall predominantly within one arm of the study and will have systematically different outcomes from the wider population. Neonates with missing key background data (birthweight, sex or gestational age) or data for the primary outcome will also be excluded.

Intervention

For the comparative study the intervention of interest will be early PN. We will compare outcomes between two groups *'PN'* and '*No PN'*. The *PN* group will comprise eligible infants who received PN at any point during the first seven days following birth. Receipt of PN is defined as receiving any volume, of any type of PN (standardised or tailor-made), by any route (peripheral intravenous cannula or central venous catheter) for any duration. The *No PN* group will comprise eligible infants who did not receive any PN in the first seven days after birth.

Sample size

The descriptive study will include data from approximately 450,000 infants.

For the comparative study we have calculated that 12,000 neonates are required in each group (*PN* and *No PN*) to have 90% power to detect (with two-sided significance of 5%) an absolute difference in survival to discharge of 1.3% between the groups. We calculated the absolute difference expected using a baseline mortality rate of 3.4% (26) and an odds ratio of 0.73 for early vs. late PN suggested by previous research (9).

Around 6,000 neonates are born between 30^{+0} and 32^{+6} weeks postmenstrual age each year in England, Wales and Scotland. Thus over the five year study period we anticipate having 13,500 neonates in the *PN* group and 16,500 in the *No PN* group.

Outcomes

For peer review only

For the descriptive study the primary outcome will be any use of PN in the first seven postnatal days. For the comparative study the primary outcome will be survival to discharge home; defined as recorded as alive at final neonatal unit discharge. Secondary outcomes for the comparative study will be the other components of the neonatal core outcomes set (27):

- Late Onset Sepsis; defined in line with the Royal College of Paediatrics and Child Health National Neonatal Audit Programme (NNAP) definition "pure growth of a pathogen from blood" or "pure growth of a skin commensal" or a "mixed growth" after the first 72 hours of life (28)
- Necrotising enterocolitis; defined in line with the Royal College of Paediatrics and Child Health National Neonatal Audit Programme (NNAP) definition. Necrotising enterocolitis may be diagnosed at surgery, post-mortem or on the basis of the following clinical and radiographic signs: at least one clinical feature from (i) Bilious gastric aspirate or emesis (ii) Abdominal distension (iii) Occult or gross blood in stool (no fissure) and at least one radiographic feature from: (i) Pneumatosis (ii) Hepato-biliary gas (iii) Pneumoperitoneum (28). As this definition was introduced in 2016 for cases where data has not been recorded this alternative definition will be used; NEC defined as a recorded diagnosis of NEC in an infant that received at least five consecutive days of antibiotics while kept nil by mouth (29)
- Brain injury on imaging; defined as documented diagnosis of intraventricular haemorrhage (grade 3-4) (30) or cystic periventricular leucomalacia
- Retinopathy of prematurity; defined as a record of any retinopathy of prematurity on routine screening in the National Neonatal Dataset "retinopathy of prematurity ad-hoc form"
- Bronchopulmonary dysplasia; defined in line with the Royal College of Paediatrics and Child Health National Neonatal Audit Programme (NNAP)

For peer review only

definition of severe bronchopulmonary dysplasia “receiving respiratory support at 36 weeks corrected gestational age” (28)

- Need for surgical procedures; defined as any record of surgical procedure during the neonatal admission
- Seizures; defined as any recorded diagnosis of seizures or seizure disorder
- Growth; weight and head circumference, and standard deviation score (SDS) of the weight and head circumference for postmenstrual age at discharge; weight velocity and head circumference velocity, and change in standard deviation score (SDS) of the weight and head circumference for postmenstrual age from birth to discharge
- Blindness; defined as an answer of Yes to the question “Does this child have a visual impairment?” at 2 years of age (28)
- Deafness; defined as an answer of Yes to the question “Does this child have a hearing impairment?” at 2 years of age (28)
- Ability to walk; defined as an answer of Yes to the question “Is this child unable to walk without assistance?” at 2 years of age (28)

The components of the core outcomes set quality of life, gross motor ability and cognitive ability will not be reported as relevant data are not captured in the NNRD.

Data analysis plan

In the descriptive study we will describe the characteristics of neonates that receive PN in the first seven postnatal days. Infants will be grouped according to gestational age at birth (using WHO definitions) (31), birth weight (using the WHO classification) (32), by year of birth and by geographical region (at the level of neonatal network). Rates of PN use will be compared between different groups using the Chi-squared test.

For peer review only

In the comparative study we will use propensity matching to minimise bias and confounding. We will ensure the two groups are as closely matched as possible except for the exposure of interest, administration of PN in the first week. Infants will be matched on gestational age at birth (in bands: 30^{+0} to 30^{+6}, 31^{+0} to 31^{+6}, 32^{+0} to 32^{+6}), low birth weight (treated as a dichotomous variable: <10th centile on the UK-WHO growth chart (33), or ≥10th centile) and propensity score. The propensity scores will be divided into propensity groups by the method of splitting proposed by Imbens and Rubin, with appropriate trimming of babies with unusually high or low scores (34). The propensity model will include maternal factors, infant factors at birth, infant factors occurring on the first day of birth (preceding the decision to administer PN) and organisational factors. A full list of background variables to be included in the propensity score can be found in eText 1.

We will calculate absolute risk differences and odds ratios for the pre-specified, dichotomous outcomes. All p values will be two-sided. We will use the Holm-Bonferroni method (35) when analysing secondary outcomes to avoid erroneous inferences due to the risk of false positives in multiple comparisons.

Subgroup analyses

As part of the descriptive survey we will compare PN use in infants of different gestational ages, different birth weights, in different geographical regions at neonatal network level (22), and compare how PN use has changed over the 5 year period.

We will undertake a sensitivity analysis of the comparative study to explore the possibility that an unobserved variable explains any effect size seen to minimise the risk of findings due to confounding. We will construct a dichotomous variable to stack the odds against the superior treatment option and then compare this to the observed background variables to explore whether it is plausible that such an unobserved variable exists (36).

For peer review only

Patient and public involvement

This project was planned and designed with input from two parents of preterm infants who had experience of PN. This project will measure outcomes that we identified as most important to over 400 stakeholders with experience of neonatal care, including former patients and parents (27). Our study addresses three research priorities identified by the James Lind Alliance priority setting partnership for preterm birth (37), namely prevention of infection, lung damage and necrotising enterocolitis.

Ethics and dissemination

This study will only use de-identified data already held within the NNRD. The NNRD is UK Research Ethics Committee approved (REC Reference: 16/LO/1093) and Confidentiality Advisory Group approved (ECC 8-05(f/2010)) and all data is stored on NHS servers. Parents can opt out of their baby's data being held within the NNRD. Study specific REC approval and Health Research Authority and Health and Care Research Wales approval was obtained (18/NI/0214).

The results of this study will be presented at academic conferences and published in a peer-reviewed scientific journal. Bliss will aid dissemination in an appropriate form online and via social media.

Discussion

Uncertainty surrounds the use of PN in neonatal patients. Previous surveys of practice show that the use of PN in neonates is variable both nationally and internationally (17, 38, 39). It is not known how PN is used in neonatal units in England, Scotland and Wales nor how this has changed over time (40), and the gestational age at which the nutritional benefits of early PN outweigh the risks in moderately preterm babies is unknown. Clinical

For peer review only

practice may vary due to the uncertainty surrounding the risks and benefits for individual babies; our study will give clinicians more evidence on which to base decisions about early use of PN.

The major limitation of any observational study is that the intervention is not randomly assigned and any differences in outcomes may be explained by confounding. We will address this issue using propensity score matching to generate two matched cohorts, analogous to the random allocation that would occur in a controlled trial. The NNRD contains comprehensive background data on both the infants and their mothers which will be included in the propensity score; these data have been demonstrated to be sufficiently robust for research purposes (23, 24). This means that we can ensure that any difference in outcomes seen is likely to be due to the exposure of interest rather than confounders. As propensity score matching only ensures that measured variables are balanced, we will undertake a sensitivity analysis to explore whether confounding due to an unmeasured variable is likely. Further strengths of this study are that data were entered by clinicians during routine care and so should not be subject to recall bias. The NNRD covers all neonatal units in England, Walesa and Scotland, hence "recruitment bias" due to incomplete population coverage is eliminated and findings will be generalisable to other populations.

In this work we will describe rates of PN use in neonatal units across England, Scotland and Wales for the first time. We will also describe the rates of important neonatal outcomes in moderately preterm neonates who receive early PN. This data will allow future randomised controlled trials to calculate expected PN exposure rates so that recruitment can be planned and provide outcome rates to allow accurate sample size calculation. Identifying whether moderately preterm neonates benefit from early PN will guide practice and also inform future research. At present there is uncertainty around optimal use of PN in preterm infants, this protocol describes a database study that is the first step in addressing this problem.

For peer review only

15

For peer review only

ABBREVIATIONS

COIN	Core Outcomes in Neonatology
NICU	Neonatal Intensive Care Unit
NDS	Neonatal Data Set
NHS	National Health Service
NNAP	National Neonatal Audit Project
NNRD	National Neonatal Research Database
REC	Research Ethics Committee
RCPCH	Royal College of Paediatrics and Child Health
SCBU	Special Care Baby Unit

For peer review only

DECLARATIONS

Consent for publication

Not applicable

Availability of data and material

Not applicable

Competing interests

JW has received support from Chiesi Pharmaceuticals to attend an educational conference and has received a research grant from Mason Medical Research Foundation.

SU has received funding from the National Institute of Health Research, the Department of Health and Prolacta Life Sciences. SU has been on the Advisory Board of Fresenius Kabi and received honoraria and travel expenses for speaking at study days organised by Fresenius Kabi. SU is a member of the National Institute for Health and Care Excellence Parenteral Nutrition Guideline Development Committee. NM is Director of the Neonatal Data Analysis Unit at Imperial College London. In the last five years NM has served on the Board of Trustees of the Royal College of Paediatrics and Child Health, David Harvey Trust, Medical Women's Federation and Medact; and is a member of the Nestle Scientific Advisory Board. NM has received research grants from the British Heart Foundation, Medical Research Council, National Institute of Health Research, Westminster Research Fund, Collaboration for Leadership in Applied Health and Care Northwest London, Healthcare Quality Improvement Partnership, Bliss, Prolacta Life Sciences, Chiesi, Shire and HCA International; travel and accommodation expenses from, Nutricia, Prolacta, Nestle and Chiesi; honoraria from Ferring Pharmaceuticals and Alexion Pharmaceuticals for contributions to expert advisory boards, and Chiesi for contributing to a lecture programme.

For peer review only

CG is part of an international team developing reporting guidance (a CONSORT extension) for clinical trials using cohorts and routinely collected health data. He has received support from Chiesi Pharmaceuticals to attend an educational conference; in the past 5 years he been investigator on received research grants from Medical Research Council, National Institute of Health Research, Canadian Institute of Health Research, Department of Health in England, Mason Medical Research Foundation, Westminster Medical School Research Trust and Chiesi Pharmaceuticals.

Funding

The funding for creating and maintaining the NNRD is from unrestricted funding awarded to NM. This includes costs involved in data transfer, storage, cleaning, merging, administration and regulatory approvals. The extraction of study data from the NNRD and analysis for this study is funded through a Mason Medical Research Fellowship awarded to JW. CG is funded by the United Kingdom Medical Research Council (MRC) through a Clinician Scientist Fellowship award. The MRC and Mason Medical Research Foundation were not involved in the design of the study, collection, analysis, and interpretation of data or in writing the manuscript.

Authors' contributions

JW, CG and NM conceived this project. JW, CG and NL planned the statistical analyses. The first draft of the manuscript was written by JW and revised by NM; CG, SU, NL and NM edited and reviewed the manuscript. It was approved by JW, CG, NL, SU and NM.

Acknowledgements

We wish to thank Angela Richard-Löndt and Laura Noakes, parents of preterm infants, for their support in developing this research project and BLISS for their input and support developing the dissemination plan.

For peer review only

19

REFERENCES

1. Fanaro S. Feeding intolerance in the preterm infant. Early Hum Dev. 2013;89:S13-S20.
2. Christensen RD, Henry E, Wiedmeier SE, Burnett J, Lambert DK. Identifying patients, on the first day of life, at high-risk of developing parenteral nutrition-associated liver disease. Journal of perinatology : official journal of the California Perinatal Association. 2007;27(5):284-90. Epub 2007/03/09.
3. Ksiazyk J, Swietlinski J, Brozek G, Musialik-Swietlinska E, Migdal M, Zejda JE. [Parenteral nutrition in neonatal units: a survey of current practices in Poland]. Medycyna wieku rozwojowego. 2008;12(4 Pt 1):899-904. Epub 2008/01/01. Zywienie pozajelitowe w oddzialach neonatologii w Polsce - analiza stosowanych praktyk.
4. British Association of Perinatal Medicine. The Provision of Parenteral Nutrition within Neonatal Services - A Framework for Practice. [Guideline] 2016 [updated April 2016; cited 2017 25/05/2017]; Available from: http://www.bapm.org/publications/documents/guidelines/Parenteral%20Nutrition%20April%202016.pdf.
5. Trivedi A, Sinn JKH. Early versus late administration of amino acids in preterm infants receiving parenteral nutrition. Cochrane Db Syst Rev. 2013(7).
6. Moyses HE, Johnson MJ, Leaf AA, Cornelius VR. Early parenteral nutrition and growth outcomes in preterm infants: a systematic review and meta-analysis. The American journal of clinical nutrition. 2013;97(4):816-26. Epub 2013/03/01.
7. Zingg W, Tomaske M, Martin M. Risk of parenteral nutrition in neonates--an overview. Nutrients. 2012;4(10):1490-503. Epub 2012/12/04.
8. Casaer MP, Mesotten D, Hermans G, Wouters PJ, Schetz M, Meyfroidt G, et al. Early versus late parenteral nutrition in critically ill adults. The New England journal of medicine. 2011;365(6):506-17. Epub 2011/07/01.
9. Fivez T, Kerklaan D, Mesotten D, Verbruggen S, Wouters PJ, Vanhorebeek I, et al. Early versus Late Parenteral Nutrition in Critically Ill Children. The New England journal of medicine. 2016;374(12):1111-22. Epub 2016/03/16.
10. van Puffelen E, Vanhorebeek I, Joosten KFM, Wouters PJ, Van den Berghe G, Verbruggen S. Early versus late parenteral nutrition in critically ill, term neonates: a preplanned secondary subgroup analysis of the PEPaNIC multicentre, randomised controlled trial. The Lancet Child & adolescent health. 2018;2(7):505-15. Epub 2018/09/01.
11. Heird WC, Gomez MR. Parenteral nutrition in low-birth-weight infants. Annu Rev Nutr. 1996;16:471-99.
12. Simmer K, Rao SC. Early introduction of lipids to parenterally-fed preterm infants. The Cochrane database of systematic reviews. 2005(2):CD005256. Epub 2005/04/23.
13. Uthaya S, Liu X, Babalis D, Dore CJ, Warwick J, Bell J, et al. Nutritional Evaluation and Optimisation in Neonates: a randomized, double-blind controlled trial of amino acid regimen and intravenous lipid composition in preterm parenteral nutrition. The American journal of clinical nutrition. 2016;103(6):1443-52. Epub 2016/04/22.
14. Tan MJ, Cooke RW. Improving head growth in very preterm infants - a randomised controlled trial I: neonatal outcomes. Arch Dis Child-Fetal. 2008;93(5):F337-F41.
15. Vlaardingerbroek H, Vermeulen MJ, Rook D, van den Akker CHP, Dorst K, Wattimena JL, et al. Safety and Efficacy of Early Parenteral Lipid and High-Dose Amino Acid Administration to Very Low Birth Weight Infants. Journal of Pediatrics. 2013;163(3):638-+.
16. Lanman JT, Guy LP, Dancis J. Retrolental fibroplasia and oxygen therapy. Journal of the American Medical Association. 1954;155(3):223-6. Epub 1954/05/15.
17. Lapillonne A, Kermorvant-Duchemin E. A systematic review of practice surveys on parenteral nutrition for preterm infants. The Journal of nutrition. 2013;143(12 Suppl):2061S-5S. Epub 2013/10/11.
18. Mason DG, Puntis JW, McCormick K, Smith N. Parenteral nutrition for neonates and children: a mixed bag. Arch Dis Child. 2011;96(3):209-10. Epub 2010/09/21.

For peer review only - http://bmjopen.bmj.com/site/about/guidelines.xhtml

19. Gale C, Morris I, NDAU NDAU. The UK National Neonatal Research Database: using neonatal data for research, quality improvement and more. Arch Dis Childhood-E. 2016;101(4):216-8.
20. Spencer A, Modi N. National neonatal data to support specialist care and improve infant outcomes. Archives of disease in childhood Fetal and neonatal edition. 2013;98(2):F175-80. Epub 2012/01/05.
21. NHS Digital. National Neonatal Data Set. 2017 [12/07/2017]; Available from: http://www.datadictionary.nhs.uk/data_dictionary/messages/clinical_data_sets/data_sets/national_neonatal_data_set/national_neonatal_data_set_-_episodic_and_daily_care_fr.asp?shownav=1.
22. Gale C, Santhakumaran S, Nagarajan S, Statnikov Y, Modi N, Unit NDA, et al. Impact of managed clinical networks on NHS specialist neonatal services in England: population based study. British Medical Journal. 2012;344.
23. Battersby C, Statnikov Y, Santhakumaran S, Gray D, Modi N, Costeloe K, et al. The United Kingdom National Neonatal Research Database: A validation study. PloS one. 2018;13(8).
24. Battersby C, Longford N, Mandalia S, Costeloe K, Modi N. Incidence and enteral feed antecedents of severe neonatal necrotising enterocolitis across neonatal networks in England, 2012-13: a whole-population surveillance study. The lancet Gastroenterology & hepatology. 2017;2(1):43-51. Epub 2017/04/14.
25. Fraser LK, Miller M, Hain R, Norman P, Aldridge J, McKinney PA, et al. Rising national prevalence of life-limiting conditions in children in England. Pediatrics. 2012;129(4):e923-9. Epub 2012/03/14.
26. Manktelow BN, Smith LK, Prunet C, Smith PW, Boby T, Hyman-Taylor P, et al. MBRRACE-UK Perinatal Surveillance Report: UK Perinatal Deaths for Births from January to December 2015. Leicester: The Infant Mortality and Morbidity Studies, Department of Health Sciences, University of Leicester, 2017.
27. Webbe J, Brunton G, Ali S, Duffy JM, Modi N, Gale C. Developing, implementing and disseminating a core outcome set for neonatal medicine. BMJ Paediatrics Open. 2017;1(1).
28. Royal College of Paediatrics and Child Health. National Neonatal Audit Programme A guide to the 2018 audit measures. Royal College of Paediatrics and Child Health, 2017.
29. Battersby C, Longford N, Patel M, Selby E, Ojha S, Dorling J, et al. Study protocol: optimising newborn nutrition during and after neonatal therapeutic hypothermia in the United Kingdom: observational study of routinely collected data using propensity matching. BMJ open. 2018;8(10):e026739. Epub 2018/10/26.
30. Papile LA, Burstein J, Burstein R, Koffler H. Incidence and Evolution of Subependymal and Intra-Ventricular Hemorrhage - Study of Infants with Birth Weights Less Than 1,500 Gm. Journal of Pediatrics. 1978;92(4):529-34.
31. Blencowe H, Cousens S, Chou D, Oestergaard M, Say L, Moller AB, et al. Born Too Soon: The global epidemiology of 15 million preterm births. Reprod Health. 2013;10.
32. World Health Organization. International Statistical Classification of Diseases and Related Health Problems 10th Revision. 2016.
33. Onis M, Onyango A, Borghi E, Siyam A, Pinol A, Garza C, et al. Enrolment and baseline characteristics in the WHO Multicentre Growth Reference Study. Acta Paediatrica. 2006;95:7-15.
34. Imbens GW, Rubin DB. Causal Inference for Statistics, Social, and Biomedical Sciences: An Introduction. Cambridge: Cambridge University Press; 2015.
35. Holm S. A Simple Sequentially Rejective Multiple Test Procedure. Scand J Stat. 1979;6(2):65-70.
36. Rosenbaum PR, Rubin DB. Assessing Sensitivity to an Unobserved Binary Covariate in an Observational Study with Binary Outcome. J Roy Stat Soc B Met. 1983;45(2):212-8.
37. Duley L, Uhm S, Oliver S. Top 15 UK research priorities for preterm birth. Lancet. 2014;383(9934):2041-2. Epub 2014/06/17.

38. Lapillonne A, Carnielli VP, Embleton ND, Mihatsch W. Quality of newborn care: adherence to guidelines for parenteral nutrition in preterm infants in four European countries. BMJ open. 2013;3(9):e003478. Epub 2013/09/21.
39. Ziegler EE, Thureen PJ, Carlson SJ. Aggressive nutrition of the very low birthweight infant. Clinics in perinatology. 2002;29(2):225-44. Epub 2002/08/10.
40. Koletzko B, Goulet O, Hunt J, Krohn K, Shamir R. 1. Guidelines on Paediatric Parenteral Nutrition of the European Society of Paediatric Gastroenterology, Hepatology and Nutrition (ESPGHAN) and the European Society for Clinical Nutrition and Metabolism (ESPEN), Supported by the European Society of Paediatric Research (ESPR). Journal of pediatric gastroenterology and nutrition. 2005;41 Suppl 2:S1-87. Epub 2005/10/29.

For peer review only

Online only supplemental material

eTable 1: Exclusion criteria for comparative study: list of major congenital gastrointestinal malformations
eTable 2: Exclusion criteria for comparative study: list of life-limiting conditions or conditions requiring surgery in the neonatal period
eText 1: Data fields for extraction from NNRD

For peer review only

eTable 1: Exclusion criteria for comparative study: list of major congenital gastrointestinal malformations

Cleverme d code	ICD-10 code	Diagnosis
10741	Q39.0	Oesophageal atresia without distal fistula
16195	Q39.0	Atresia of oesophagus without fistula
10740	Q39.1	Oesophageal atresia with distal tracheo-oesophageal fistula
16196	Q39.1	Atresia of oesophagus with tracheo-oesophageal fistula (TOF)
16197	Q39.2	Congenital tracheo-oesophageal fistula without atresia (TOF)
10273	Q39.3	Congenital stenosis of the oesophagus
16198	Q39.3	Congenital stenosis and stricture of oesophagus
16199	Q39.4	Oesophageal web
10358	Q41.0	Duodenal atresia / stenosis / web (specify)
16212	Q41.0	Congenital absence, atresia and stenosis of duodenum
16213	Q41.0DA	Duodenal atresia / stenosis
10605	Q41.1	Jejunal atresia / stenosis (specify)
16214	Q41.1JA	Jejunal atresia / stenosis
10541	Q41.2	Ileal atresia / stenosis (specify)
16215	Q41.2	Congenital absence, atresia and stenosis of ileum
16216	Q41.2IA	Ileal atresia / stenosis
16217	Q41.X	Congenital absence, atresia and stenosis of small intestine
16218	Q42.0	Congenital absence, atresia and stenosis of rectum with fistula
10496	Q42.00	High anorectal anomaly with rectourethral fistula
10497	Q42.01	High anorectal anomaly with rectovesical fistula
10498	Q42.02	High anorectal anomaly with rectovulval fistula
10495	Q42.03	High anorectal anomaly with rectocutaneous fistula
10494	Q42.04	High anorectal anomaly with rectocloacal fistula
10493	Q42.08	High anorectal anomaly with fistula (specify)
10499	Q42.1	High anorectal anomaly without fistula
16219	Q42.1	Congenital absence, atresia and stenosis of rectum without fistula
16220	Q42.2	Congenital absence, atresia and stenosis of anus with fistula
10636	Q42.20	Low anorectal anomaly with anocutaneous fistula
10637	Q42.21	Low anorectal anomaly with anovestibular fistula
10638	Q42.28	Low anorectal anomaly with fistula (other specify)
10639	Q42.3	Low anorectal anomaly without fistula
16221	Q42.3	Congenital absence, atresia and stenosis of anus without fistula
10240	Q42.31	Congenital anal stenosis
16222	Q42.8	Congenital absence, atresia and stenosis of anus of other parts of large intestine
16223	Q429	Congenital absence, atresia and stenosis of anus of large intestine, part unspecified
16224	Q42X	Congenital absence, atresia and stenosis of large intestine

For peer review only

16235	Q43.7	Persistent cloaca

For peer review only

eTable 2: Exclusion criteria for comparative study: list of life-limiting conditions or conditions requiring surgery in the neonatal period

Clevermed code	ICD-10 code	Diagnosis
15890	Q00.0	Anencephaly
15891	Q00.1	Craniorachischisis
15892	Q00.2	Iniencephaly
15893	Q00.X	Anencephaly and similar malformations
15894	Q01.0	Frontal encephalocele
15895	Q01.1	Nasofrontal encephalocele
15896	Q01.2	Occipital encephalocele
15897	Q01.8	Encephalocele of other sites
15898	Q01.9	Encephalocele (unknown or unspecified cause)
15899	Q01.X	Encephalocele
15918	Q04.2	Holoprosencephaly
15926	Q05.0	Cervical spina bifida with hydrocephalus
15927	Q05.1	Thoracic spina bifida with hydrocephalus
15928	Q05.2	Lumbar spina bifida with hydrocephalus
15929	Q05.3	Sacral spina bifida with hydrocephalus
15930	Q05.4	(unknown or unspecified cause) spina bifida with hydrocephalus
15931	Q05.5	Cervical spina bifida without hydrocephalus
15932	Q05.6	Thoracic spina bifida without hydrocephalus
15933	Q05.7	Lumbar spina bifida without hydrocephalus
15934	Q05.8	Sacral spina bifida without hydrocephalus
15935	Q05.9	Spina bifida (unknown or unspecified cause)
10986	Q05.9a	Spina bifida
10704	Q05.9b	Myelomeningocele (specify site)
15936	Q05.X	Spina bifida
16024	Q20.0	Common arterial trunk (Truncus malformation)
10356	Q20.1	Double outlet right ventricle (DORV)
16025	Q20.1	Double outlet right ventricle (DORV)
16026	Q20.2	Double outlet left ventricle (DOLV)
11070	Q20.3	Transposition of the great vessels (TGA)
16027	Q20.3	Transposition great arteries (TGA)
16028	Q20.4	Double inlet ventricle (DILV)
16029	Q20.5	Discordant atrioventricular connection
16030	Q20.6	Isomerism of atrial appendages
16031	Q20.8	Other cong malforms of cardiac chambers and connections
16032	Q20.9	Cong malforms of cardiac chambers and connections unspec
16033	Q20.X	Congenital malformations of cardiac chambers and connections
16035	Q20.91	Atrium single

For peer review only

16036	Q20.92	Ventricle single
10097	Q21.2	Atrio-ventricular septal defect (AVSD)
16039	Q21.2	Atrioventricular septal defect (AVSD)
11043	Q21.3	Tetralogy of Fallot
16040	Q21.3	Tetralogy of Fallot
16045	Q22.0	Pulmonary valve atresia
16046	Q22.1	Congenital pulmonary valve stenosis
16047	Q22.2	Congenital pulmonary valve insufficiency
16048	Q22.3	Other congenital malformations of pulmonary valve
16049	Q22.4	Congenital tricuspid atresia / stenosis
16050	Q22.5	Ebstein's anomaly
16051	Q22.6	Hypoplastic right heart syndrome
16052	Q22.8	Other congenital malformations of tricuspid valve
16053	Q22.9	Congenital malformation of tricuspid valve (unknown or unspecified cause)
16054	Q22.X	Congenital malformations of pulmonary and tricuspid valves
16055	Q23.0	Congenital stenosis of aortic valve (AS)
16056	Q23.1	Congenital insufficiency of aortic valve
16057	Q23.2	Congenital mitral stenosis (MS)
16058	Q23.3	Mitral atresia
16059	Q23.4	Hypoplastic left heart syndrome (HLH)
16060	Q23.8	Other congenital malformations of aortic and mitral valves
16061	Q23.9	Congenital malformation of aortic and mitral valves unspec
16062	Q23.X	Congenital malformations of aortic and mitral valves
16079	Q25.1	Coarctation of aorta
10227	Q25.19	Coarctation of the aorta
16080	Q25.2	Hypoplasia of aortic arch
16081	Q25.3	Stenosis of aorta (AS)
16082	Q25.4	Malformation of aorta
16083	Q25.5	Atresia of pulmonary artery
16084	Q25.6	Stenosis of pulmonary artery (PS)
16086	Q25.8	Other congenital malformations of great arteries
16087	Q25.8	Transposition of the great vessels (TGA)
11057	Q26.2	Total anomylous pulmonary venous drainage (TAPVD)
16092	Q26.2	Total anomalous pulmonary venous connection (TAPVD)
16154	Q33.6	Hypoplasia and dysplasia of lung
16241	Q44.2	Atresia of bile ducts
10123	Q60.1	Bilateral renal agenesis
16318	Q60.1B	Renal agenesis, bilateral
16324	Q60.6	Potter's syndrome
16327	Q61.1	Polycystic kidney, infantile type
10100	Q61.1a	Autosomal recessive polycystic kidney - infantile

For peer review only

10367	Q64.1	Ectopia vesicae
16356	Q64.1	Exstrophy of urinary bladder
10854	Q64.2	Posterior urethral valves (PUV)
16357	Q64.2	Congenital posterior urethral valves (PUV)
16360	Q64.5	Congenital absence of bladder and urethra
10008	Q64.5a	Absence of bladder
10236	Q64.5b	Congenital absence of urethra
16475	Q77.1	Thanatophoric short stature
10246	Q79.0	Congenital diaphragmatic hernia
10490	Q79.0	Hernia into the cord
16495	Q79.0	Congenital diaphragmatic hernia
16496	Q79.1A	Aplasia of diaphragm
16497	Q79.1E	Eventration of diaphragm
16498	Q79.2	Exomphalos
10395	Q79.2	Exomphalos
16499	Q79.3	Gastroschisis
16589	Q90.0	Trisomy 21, meiotic nondisjunction
16590	Q90.1	Trisomy 21, mosaicism (mitotic nondisjunction)
16591	Q90.2	Trisomy 21, translocation
16592	Q90.9	Down's syndrome (unknown or unspecified cause)
16593	Q90.X	Down's syndrome
16594	Q91.0	Trisomy 18, meiotic nondisjunction
16595	Q91.1	Trisomy 18, mosaicism (mitotic nondisjunction)
16596	Q91.2	Trisomy 18, translocation
16597	Q91.3	Edwards' syndrome (unknown or unspecified cause)
16598	Q91.4	Trisomy 13, meiotic nondisjunction
16599	Q91.5	Trisomy 13, mosaicism (mitotic nondisjunction)
16600	Q91.6	Trisomy 13, translocation
16601	Q91.7	Patau's syndrome (unknown or unspecified cause)
16602	Q91.X	Edwards' syndrome and Patau's syndrome

For peer review only

eText 1: Data fields for extraction from NNRD

**Treatment**	
**Variable**	**Data items**
Parenteral nutrition	**PN group** defined as<br>Any of the following items entered in the 'Daily Care Fluids' and 'Feeding' during first 7 days:<br>• Y entry for PARENTERAL NUTRITION RECEIVED INDICATOR<br>Or<br>The following drug code entered in the Daily care medication during first 7 days:<br>• 1010238 Total parenteral nutrition<br><br>**No PN group** defined as<br>All other babies

**Background variables for matching**	
**Variable**	**Data items**
Gestational age at birth	**30^{+0} to 30^{+6} group** defined as<br>Any of the following items entered in the GESTATION LENGTH (AT DELIVERY):<br>• 30^{+0}, 30^{+1}, 30^{+2}, 30^{+3}, 30^{+4}, 30^{+5}, 30^{+6}<br><br>**31^{+0} to 31^{+6} group** defined as<br>Any of the following items entered in the GESTATION LENGTH (AT DELIVERY):<br>• 31^{+0}, 31^{+1}, 31^{+2}, 31^{+3}, 31^{+4}, 31^{+5}, 31^{+6}<br><br>**32^{+0} to 32^{+6} group** defined as<br>Any of the following items entered in the GESTATION LENGTH (AT DELIVERY):<br>• 32^{+0}, 32^{+1}, 32^{+2}, 32^{+3}, 32^{+4}, 32^{+5}, 32^{+6}
Low birth weight	**Low birth weight group** defined as<br>Any result entered in the BIRTH WEIGHT which is below the 10th centile on the UK-WHO growth chart<br><br>**Normal birth weight group** defined as<br>All other babies

**Background matching for propensity score matching**	
**Variable**	**Data items**
Sex	Data will be extracted from PERSON PHENOTYPIC SEX<br>• Categorical: 1 Male / 2 Female / 9 Indeterminate (unable to be classified as either male or female)
Multiplicity	Data will be extracted from NUMBER OF FETUSES (NOTED DURING PREGNANCY EPISODE); this excludes fetus papyraceous and fetuses reabsorbed in utero and not delivered.
Year of birth	Data will be extracted from DATE TIME OF BIRTH<br>• Continuous in one year bands
Maternal age	Data will be extracted from YEAR OF BIRTH (MOTHER)<br>• Continuous variable
Maternal diabetes	Data will be extracted from MATERNITY COMPLICATING MEDICAL DIAGNOSIS TYPE (NATIONAL NEONATAL DATA SET)<br>• Dichotomous: 08 Y/N
Maternal gestational	Data will be extracted from MATERNITY OBSTETRIC DIAGNOSIS

For peer review only

diabetes	TYPE (CURRENT PREGNANCY)<br>• Dichotomous: 06 Gestational diabetes mellitus Y/N
Maternal severe pre-eclampsia requiring pre-term birth	Data will be extracted from MATERNITY OBSTETRIC DIAGNOSIS TYPE (CURRENT PREGNANCY)<br>• Dichotomous: 01 Severe pre-eclampsia requiring pre-term birth Y/N
Maternal severe pre-eclampsia	Data will be extracted from MATERNITY OBSTETRIC DIAGNOSIS TYPE (CURRENT PREGNANCY)<br>• Dichotomous: 20 Severe pre-eclampsia Y/N
Maternal gestational hypertension	Data will be extracted from MATERNITY OBSTETRIC DIAGNOSIS TYPE (CURRENT PREGNANCY)<br>• Dichotomous: 07 Gestational hypertension Y/N
Maternal prolonged rupture of membranes	Data will be extracted from NUMBER OF MINUTES (BIRTH TO EVENT)<br>• Continuous variable
Maternal suspected chorioamnionitis	Data will be extracted from SIGNIFICANT MATERNAL PYREXIA IN LABOUR INDICATOR or INTRAPARTUM ANTIBIOTICS GIVEN INDICATOR<br>• Dichotomous: Suspected chorioamnionitis defined as Y in either field<br>• Dichotomous: No suspected chorioamnionitis defined as N in both fields
Maternal receipt of antenatal steroids	Data will be extracted from STEROIDS GIVEN DURING PREGNANCY TO MATURE FETAL LUNGS INDICATOR (Y/N) and ANTENATAL STEROID COURSE COMPLETION STATUS<br>• Categorical: Complete course defined as Y and 1 Complete course<br>• Categorical: Incomplete course defined as Y and 2 Incomplete course<br>• Categorical: No steroids defined as N
Maternal receipt of antenatal magnesium sulphate	Data will be extracted from MOTHER RECEIVED MAGNESIUM SULPHATE IN 24 HOURS PRIOR TO DELIVERY<br>• Dichotomous: Y/N
Infant Apgar score at 5 minutes	Data will be extracted from APGAR SCORE (5 MINUTES)<br>• Categorical: 0-10
Infant: chest compressions administered	Data will be extracted from NEONATAL RESUSCITATION METHODS (NATIONAL NEONATAL DATA SET)<br>• Dichotomous: 16 Cardiac massage (Y/N)
Infant: Emergency resuscitation drugs administered	Data will be extracted from NEONATAL RESUSCITATION METHODS (NATIONAL NEONATAL DATA SET)<br>• Dichotomous: 17 Adrenaline or 88 Any other drug (Y/N)
Infant: Intubated at resuscitation	Data will be extracted from NEONATAL RESUSCITATION METHODS (NATIONAL NEONATAL DATA SET)<br>• Dichotomous: 15 Intubation (Y/N)
Infant: Surfactant administered	Data will be extracted from SURFACTANT GIVEN INDICATOR (DURING RESUSCITATION)<br>• Dichotomous: Y/N
Infant: Umbilical cord pH	Data will be extracted from UMBILICAL CORD BLOOD PH LEVEL (ARTERIAL)<br>• Continuous: Arterial cord pH (6.00-8.00)<br>Or if unavailable use:<br>UMBILICAL CORD BLOOD PH LEVEL (VENOUS)<br>• Continuous: Venous cord pH (6.00-8.00)
Infant: Admission temperature	Data will be extracted from TEMPERATURE (ON ADMISSION TO NEONATAL CRITICAL CARE)<br>• Continuous

For peer review only

Infant: Admission mean blood pressure	Data will be extracted from MEAN ARTERIAL BLOOD PRESSURE (ON ADMISSION TO NEONATAL CRITICAL CARE)<br>• Continuous: 10-150
Infant: Admission blood glucose	Data will be extracted from BLOOD GLUCOSE CONCENTRATION (ON ADMISSION TO NEONATAL CRITICAL CARE)<br>• Continuous: 0.0-50.0
Infant: Admission heart rate	Data extracted from HEART RATE (ON ADMISSION TO NEONATAL CRITICAL CARE)<br>• Continuous: 50-350
Infant: Admission respiratory rate	Data extracted from RESPIRATORY RATE (ON ADMISSION TO NEONATAL CRITICAL CARE)<br>• Continuous: 10-200
Infant: Admission oxygen saturation	Data extracted from OXYGEN SATURATION (ON ADMISSION TO NEONATAL CRITICAL CARE)<br>• Continuous: 10-100
Infant: Surfactant administered on first day	Data extracted from SURFACTANT GIVEN INDICATOR (ON NEONATAL CRITICAL CARE DAILY CARE DATE)<br>• Continuous: Y/N
Infant: Mechanical ventilation on first day	Data extracted from RESPIRATORY SUPPORT DEVICE TYPE (NATIONAL NEONATAL DATA SET) for first day<br>• Mechanical ventilation defined as 1 Endotracheal tube<br>• No ventilation defined as any other answer
Infant: Inotropes administered on first day	Data extracted from INOTROPE INFUSION RECEIVED INDICATOR for first day<br>• Dichotomous: Y/N<br>Or DAILY CARE MEDICATION on day 1 only<br>• 500098 Dopamine<br>• 500096 Dobutamine<br>• 500056 Adrenaline<br>• 500210 Noradrenaline<br>• 500116 Hydrocortisone<br>• 1010173 Milrinone
Infant: Sepsis suspected on first day	Data extracted from DAILY CARE INFECTIONS SEPSIS SUSPECTED INDICATOR for first day<br>• Y/N
Infant: Transfer on first day	Data extracted from Admission Details SITE CODE (OF ADMITTING NEONATAL UNIT) or ORGANISATION CODE (OF ADMITTING NEONATAL UNIT) is different from Baby Demographics SITE CODE (OF ACTUAL PLACE OF DELIVERY) or ORGANISATION CODE (OF ACTUAL PLACE OF<br>DELIVERY)<br>And Baby Demographics EPISODE NUMBER is >1
Level of initial neonatal unit	Data extracted from SITE CODE (OF ACTUAL PLACE OF DELIVERY)
Neonatal network	Data extracted from SITE CODE (OF ACTUAL PLACE OF DELIVERY)

**Outcomes**	
**Variable**	**Data items**

For peer review only

Survival	Data extracted from DISCHARGE DESTINATION FROM NEONATAL CRITICAL CARE<br>• Survival defined as any of 1, 2, 4, 5, 6<br>• Died defined as code 3, Died
Late onset sepsis	NNAP definition<br>Defined from Infection Cultures (Episodic) recorded after day 3<br>• Pure growth of pathogen from blood OR Pure growth of pathogen from CSF OR Either a pure growth of a skin commensal or a mixed growth with ≥3 clinical signs at the time of blood sampling
Necrotising enterocolitis	NNAP definition<br>Defined from DISCHARGE DETAILS based on WAS NEC DIAGNOSED THIS ADMISSION answer Y<br>With at least one clinical feature from:<br>• Bilious gastric aspirate or emesis/Abdominal distension/Occult or gross blood in stool (no fissure)<br>And at least one radiographic feature from:<br>• Pneumatosis/Hepato-biliary gas/Pneumoperitoneum<br><br>Where NNAP definition not recorded (e.g. prior to 2016):<br>Defined from Daily Care Gastrointestinal at any point during neonatal unit stay:<br>• Any entry (1 or 2) for TREATMENT TYPE FOR NECROTISING ENTEROCOLITIS<br>Or: From diagnostic codes •<br>• 1010683 Necrotising enterocolitis – suspected<br>• 10708 Necrotising enterocolitis – Perforated<br>• 15809 Necrotizing enterocolitis<br><br>AND<br><br>5 or more days nil by mouth defined from the Daily Care Fluids and Feeding for a continuous period of 5 days<br>• No under ENTERAL FEED TYPE GIVEN<br>• No entry under FORMULA MILK OR MILK FORTIFIER TYPE<br>• No value OR 0 for TOTAL VOLUME OF MILK RECEIVED<br>• No entry under ENTERAL FEEDING METHOD<br>While also receiving 5 or more days of antibiotics over the same 5 days as the baby was nil by mouth<br>Defined as 5 consecutive days of any of the following Daily care medication<br>• 1010155 Benzyl Penicillin<br>• 1010158 Augmentin<br>• 1010179 Flucloxicillin<br>• 500012 Flucloxacillin<br>• 500016 Gentamicin<br>• 500072 Co-amoxiclav<br>• 500086 Co-amoxiclav<br>• 500084 Ciprofloxacin<br>• 500029 Netilmicin<br>• 500002 Amikacin<br>• 500211 Tazocin<br>• 500023 Metronidazole

For peer review only

	• 500040 Vancomycin<br>• 500007 Cefotaxime<br>• 500004 Ampicillin<br>• 500009 Cefuroxime<br>• 500008 Ceftazidime<br>• 500175 Ceftriaxone<br>• 500032 Piperacillin<br>• 500206 Oflacillin<br>• 500005 Azlocillin<br>• 1010171 Linezolid<br>• 1010271 Cefalexin<br>• 1010139 Amoxicillin<br>• 500070 Amoxicillin<br>• 500128 Meropenem<br>• 500118 Imepenem<br>• 500145 Imipenem
Brain injury on imaging	Data extracted from CRANIAL ULTRASOUND SCANS (EPISODIC)<br>Brain injury defined as:<br>• INTRAVENTRICULAR HAEMORRHAGE GRADE (RIGHT SIDE) or INTRAVENTRICULAR HAEMORRHAGE GRADE (LEFT SIDE) code 3 or 4 (Grade 3/4 intraventricular haemorrhage)<br>Or:<br>• CYSTIC PERIVENTRICULAR LEUKOMALACIA OBSERVED DURING CRANIAL ULTRASOUND SCAN INDICATOR answer Y
Retinopathy of prematurity	Data extracted from RETINOPATHY OF PREMATURITY SCREENING (EPISODIC)<br>Retinopathy of prematurity defined as:<br>• RETINOPATHY OF PREMATURITY STAGE (LEFT EYE) or RETINOPATHY OF PREMATURITY STAGE (RIGHT EYE) any code except 0 (None seen)
Bronchopulmonary dysplasia	NNAP definition<br>Significant bronchopulmonary dysplasia defined as:<br>• DAILY SUMMARY at 36^{+0} receiving any respiratory support
Need for surgical procedures	Data extracted from PROCEDURE (OPCS ON NEONATAL CRITICAL CARE DAILY CARE DATE)<br>Surgery defined as any of the following codes:<br>• 100033 Surgery for meconium ileus (von)<br>• 100076 Skin or soft tissue surgery requiring general or spinal anesthesia (Description Required<br>• 11222 Closure of small intestine/ileal perforation<br>• 11501 Laparoscopy<br>• 11904 Colostomy<br>• 11905 Ileostomy<br>• 1010826 Major surgery<br><br>Or:<br>• Daily item ANY MAJOR SURGERY TODAY answer Y
Seizures	Seizure defined as:SEIZURE OCCURRED INDICATOR<br>• Y<br>Or: DIAGNOSIS (ICD RECORDED ON DISCHARGE FROM NEONATAL CRITICAL CARE) with code<br>• 10957 Seizures<br>• 15192 Seizure disorder<br>• 15194 Seizure disorder (cause unknown)<br>• 15195 Status epilepticus

For peer review only

	• 15848 Seizures
Weight	Data extracted from Daily Care General Information PERSON WEIGHT IN GRAMS from final day
Head circumference	Data extracted from Daily Care General Information HEAD CIRCUMFERENCE IN CENTIMETRES from final day

**Long Term Outcomes**	
**Variable**	**Data items**
Blindness	Defined as an answer of Yes to the question “Does this child have a visual impairment?” on the NNAP form
Deafness	Defined as an answer of Yes to the question “Does this child have a hearing impairment?” on the NNAP form
Ability to walk	Defined as an answer of Yes to the question “Is this child unable to walk without assistance?” on the NNAP form

For peer review only

BMJ Open

Outcomes following early parenteral nutrition use in preterm neonates: Protocol for an observational study

Journal:	*BMJ Open*
Manuscript ID	bmjopen-2019-029065.R1
Article Type:	Protocol
Date Submitted by the Author:	03-Apr-2019
Complete List of Authors:	Webbe, James; Imperial College London, Section of Neonatal Medicine<br>Longford, Nicholas; Imperial College London, Section of Neonatal Medicine<br>Uthaya, Sabita; Imperial College London,<br>Modi, Neena; Imperial College London, Medicine<br>Gale, Chris; Imperial College London, Section of Neonatal Medicine;
**Primary Subject Heading**:	Paediatrics
Secondary Subject Heading:	Paediatrics
Keywords:	NEONATOLOGY, Neonatal intensive & critical care < INTENSIVE & CRITICAL CARE, Nutritional support < GASTROENTEROLOGY

SCHOLARONE™ Manuscripts

1

Outcomes following early parenteral nutrition use in preterm neonates: Protocol for an observational study

James Webbe[1], Nicholas Longford[2], Sabita Uthaya[3], Neena Modi[4], Chris Gale[5]

[1] James Webbe

Section of Neonatal Medicine, Imperial College London

London, United Kingdom.

[2] Nicholas Longford

Section of Neonatal Medicine, Imperial College London

London, United Kingdom.

[3] Sabita Uthaya

Section of Neonatal Medicine, Imperial College London

London, United Kingdom.

[4] Neena Modi

Section of Neonatal Medicine, Imperial College London

London, United Kingdom.

[5] Chris Gale, Clinical Senior Lecturer

Section of Neonatal Medicine, Chelsea and Westminster campus, Imperial College London

369 Fulham Road, London, SW10 9NX, United Kingdom.

Corresponding author:

Dr James Webbe, Clinical Research Fellow

For peer review only

Section of Neonatal Medicine, Chelsea and Westminster Campus, Imperial College London

369 Fulham Road, London, SW10 9NX, United Kingdom.

+ 44 (0)203 3155418

j.webbe@imperial.ac.uk

Manuscript word count: 2797

For peer review only

3

Abstract

Introduction

Preterm babies are among the highest users of parenteral nutrition (PN) of any patient group, but there is wide variation in commencement, duration, and composition of PN and uncertainty around which groups will benefit from early introduction. Recent studies in critically unwell adults and children suggest that harms, specifically increased rates of nosocomial infection, outweigh the benefits of early administration of PN. In this study we will describe early PN use in neonatal units in England, Wales and Scotland. We will also evaluate if this is associated with differences in important neonatal outcomes in neonates born between 30^{+0} and 32^{+6} $weeks^{+days}$ gestation.

Methods and analysis

We will use routinely collected data from all neonatal units in England, Wales and Scotland, available in the National Neonatal Research Database (NNRD). We will describe clinical practice in relation to any use of PN during the first seven postnatal days among neonates admitted to neonatal care between 1st January 2012 and 31st December 2017. We will compare outcomes in neonates born between 30^{+0} and 32^{+6} $weeks^{+days}$ gestation who did or did not receive PN in the first week after birth using a propensity score matched approach. The primary outcome will be survival to discharge home. Secondary outcomes will include components of the neonatal core outcome set: outcomes identified as important by former patients, parents, clinicians and researchers.

Regulatory approvals and dissemination

We have obtained UK National Research Ethics Committee approval for this study (Ref: 18/NI/0214). The results of this study will be presented at academic conferences; the United Kingdom charity Bliss will aid dissemination to former patients and parents.

For peer review only

Registration

ClinicalTrials.gov: NCT03767634

Registration date: 06/12/2018

Keywords

[1] Parenteral nutrition [2] Preterm neonate [3] Core outcomes set [4] Propensity score matching [5] Neonatal care [6] National Neonatal Research database

Abstract word count: 295

For peer review only

5

ARTICLE SUMMARY

Strengths and limitations of this study

- We will use routinely recorded data held in the National Neonatal Research Database (NNRD); these have been shown to be complete and of high quality
- The NNRD covers the entire neonatal unit population of England, Wales and Scotland and thus provides power to explore associations between early PN use and outcomes
- As a retrospective, observational study results will be vulnerable to confounding
- The NNRD contains a large number of variables that will assist in propensity score matching to form well-balanced groups, diminishing potential confounding
- We will focus on core outcomes identified as important by former patients and parents as well as clinicians and researchers

For peer review only

Introduction

Preterm birth abruptly ends the transplacental transfer of nutrients essential for fetal growth and development. Providing adequate nutrition is an essential component of neonatal care but very preterm infants often have difficulty in tolerating adequate volumes of milk immediately after birth (1). To meet their nutritional needs such infants are commonly given supplemental parenteral nutrition (PN). Preterm babies are among the highest PN users of all NHS patients but clinical practice is variable both nationally and internationally: some neonatal units in high-income countries are reported to provide PN to around 70% of neonatal admissions (2), while others report using no PN (3). In the United Kingdom national guidelines recommend administration of PN to all babies born below 30 weeks gestational age (4), however exact rates of PN use in UK neonatal units are not known. When considering the efficacy of PN use in neonates, although the impact of early PN on nitrogen balance is known (5), the effects on survival, growth and neurodevelopment are less clear (5, 6). Despite widespread use, the impact of administration of PN on key outcomes has not been evaluated in randomised, controlled neonatal trials powered for clinical end-points.

Parenteral nutrition carries well established risks, of which the most serious and common is blood stream infection (7). There is a growing body of evidence that use of PN in critically unwell adults (8) and children (9) within the first seven days of admission to an intensive care unit is associated with worse outcomes. Furthermore, a subgroup analysis of a paediatric intensive care unit population limited to term neonates less than 28 days old showed an increase in nosocomial infection with early PN use (10). These studies raise uncertainty over the balance of risks and benefits of PN administration in the early postnatal period. It is generally accepted that PN is likely to be beneficial in extremely preterm neonates where postnatal nutrient deficits are most severe and prolonged, but in moderately preterm neonates the effect of PN use on neonatal survival or other key outcomes has never

For peer review only

been conclusively demonstrated (5, 11, 12). The largest randomised trials to date (13-15) have recruited fewer than 100 babies to each interventional arm and were therefore only powered to detect very large (10%) absolute risk differences in mortality or other outcomes. Neonates are vulnerable to unanticipated treatment effects across different organ systems (16), therefore it is important to show that PN does not adversely impact any important neonatal outcomes in addition to the nutrition outcomes commonly studied in PN trials. The scant evidence-base to inform use is reflected in variation in timing of commencement, duration of use and composition of PN within and between countries (17, 18).

This protocol describes how we will approach the following research questions:

- What is the pattern of PN use in neonatal units in England, Scotland and Wales in the first seven postnatal days?
- In neonates born between 30^{+0} and 32^{+6} weeks gestational age, is PN use in the first seven postnatal days, compared with no use of PN, associated with altered survival to discharge home?
- For neonates born between 30^{+0} and 32^{+6} weeks gestational age is use of PN in the first seven postnatal days, compared with no use of PN, associated with different core neonatal outcomes?

Methods

Study design

This study contains two projects that will use data held in the National Neonatal Research Database. First we will evaluate practice, and then we will undertake a comparison of matched groups of moderately preterm babies.

Data source

For peer review only

This study will use de-identified data held in the NNRD (19). The NNRD holds data extracted from point-of-care electronic health records completed by health professionals during routine clinical care (20). The Neonatal Data Set, a defined national data standard (21) comprising approximately 450 items, is extracted and transmitted to the Neonatal Data Analysis Unit at Imperial College London. The data set includes demographic items relating to mother and baby (e.g. gestational age at birth, birthweight, maternal conditions), daily items (e.g. feeding information, administration of PN), ad hoc items (e.g. suspected infection, cranial ultrasound findings), discharge items (e.g. diagnoses during admission, weight at discharge), and two year follow up data. It contains data about which hospital a baby is born in and which neonatal network (the managed clinical networks that provide neonatal care within a geographical are) each hospital is part of (22). The NNRD holds data from all infants admitted to NHS neonatal units in England, Scotland and Wales (approximately 90,000 infants each year). In total the NNRD contains data from approximately one million infants from 2008 to the present. Since 2012 all units in England and Wales have contributed data and since 2015 all but one Scottish units have contributed, with complete coverage of Scottish neonatal units since 2018. The completeness and quality of data held in the NNRD has been shown to be high (23), making it suitable for research (24).

For peer review only

Eligibility

For the description of practice we will utilise data on all neonates born between 1st January 2012 and 31st December 2017 and admitted to a neonatal unit in England, Scotland and Wales.

For the matched comparative study we will use data on all neonates born between 30^{+0} and 32^{+6} weeks gestational age between 1st January 2012 and 31st December 2017 and admitted to a neonatal unit in England, Scotland and Wales. Neonates with major congenital gastrointestinal malformations will be excluded as they cannot be fed enterally (eTable 1). Neonates with life limiting conditions (25) or congenital conditions requiring

surgery in the neonatal period will be excluded as they will not receive standard neonatal nutritional care (eTable 2). Both groups would bias results because they will fall predominantly within one arm of the study and will have systematically different outcomes from the wider population. Neonates with missing key background data (birthweight, sex or gestational age) or data for the primary outcome will also be excluded.

Intervention

For the comparative study the intervention of interest will be early PN. We will compare outcomes between two groups *'PN'* and '*No PN'*. The *PN* group will comprise eligible infants who received PN at any point during the first seven days following birth. Receipt of PN is defined as receiving any volume, of any type of PN (standardised or tailor-made), by any route (peripheral intravenous cannula or central venous catheter) for any duration. The *No PN* group will comprise eligible infants who did not receive any PN in the first seven days after birth.

Sample size

The descriptive study will include data from approximately 450,000 infants.

For the comparative study we have calculated that 12,000 neonates are required in each group (*PN* and *No PN*) to have 90% power to detect (with two-sided significance of 5%) an absolute difference in survival to discharge of 1.3% between the groups. We calculated the absolute difference expected using a baseline mortality rate of 3.4% (26) and an odds ratio of 0.73 for early vs. late PN suggested by previous research (9).

Around 6,000 neonates are born between 30^{+0} and 32^{+6} weeks postmenstrual age each year in England, Wales and Scotland. Thus over the five year study period we will have 30,000 neonates in total. Pilot data from the NNRD suggests 45% of this group of babies will

For peer review only

receive PN so we anticipate having 13,500 neonates in the *PN* group and 16,500 in the *No PN* group.

Outcomes

For the descriptive study the primary outcome will be any use of PN in the first seven postnatal days. For the comparative study the primary outcome will be survival to discharge home; defined as recorded as alive at final neonatal unit discharge. Secondary outcomes for the comparative study will be the other components of the neonatal core outcomes set (27):

- Late Onset Sepsis; defined in line with the Royal College of Paediatrics and Child Health National Neonatal Audit Programme (NNAP) definition "pure growth of a pathogen from blood" or "pure growth of a skin commensal" or a "mixed growth" after the first 72 hours of life (28)
- Necrotising enterocolitis; defined in line with the Royal College of Paediatrics and Child Health National Neonatal Audit Programme (NNAP) definition. Necrotising enterocolitis may be diagnosed at surgery, post-mortem or on the basis of the following clinical and radiographic signs: at least one clinical feature from (i) Bilious gastric aspirate or emesis (ii) Abdominal distension (iii) Occult or gross blood in stool (no fissure) and at least one radiographic feature from: (i) Pneumatosis (ii) Hepato-biliary gas (iii) Pneumoperitoneum (28). As this definition was introduced in 2016 for cases where data has not been recorded this alternative definition will be used; NEC defined as a recorded diagnosis of NEC in an infant that received at least five consecutive days of antibiotics while kept nil by mouth (29)
- Brain injury on imaging; defined as documented diagnosis of intraventricular haemorrhage (grade 3-4) (30) or cystic periventricular leucomalacia

For peer review only

- Retinopathy of prematurity; defined as a record of any retinopathy of prematurity on routine screening in the National Neonatal Dataset “retinopathy of prematurity ad-hoc form”
- Bronchopulmonary dysplasia; defined in line with the Royal College of Paediatrics and Child Health National Neonatal Audit Programme (NNAP) definition of severe bronchopulmonary dysplasia “receiving respiratory support at 36 weeks corrected gestational age” (28)
- Need for surgical procedures; defined as any record of surgical procedure during the neonatal admission
- Seizures; defined as any recorded diagnosis of seizures or seizure disorder
- Growth; weight and head circumference, and standard deviation score (SDS) of the weight and head circumference for postmenstrual age at discharge; weight velocity and head circumference velocity, and change in standard deviation score (SDS) of the weight and head circumference for postmenstrual age from birth to discharge
- Blindness; defined as an answer of Yes to the question “Does this child have a visual impairment?” at 2 years of age (28)
- Deafness; defined as an answer of Yes to the question “Does this child have a hearing impairment?” at 2 years of age (28)
- Ability to walk; defined as an answer of Yes to the question “Is this child unable to walk without assistance?” at 2 years of age (28)

The components of the core outcomes set quality of life, gross motor ability and cognitive ability will not be reported as relevant data are not captured in the NNRD.

Data analysis plan

In the descriptive study we will describe the characteristics of neonates that receive PN in the first seven postnatal days. Infants will be grouped according to gestational age at birth

For peer review only

(using WHO definitions) (31), birth weight (using the WHO classification) (32), by year of birth and by geographical region (at the level of neonatal network). Rates of PN use will be compared between different groups using the Chi-squared test.

In the comparative study we will use propensity matching to minimise bias and confounding. We will ensure the two groups are as closely matched as possible except for the exposure of interest, administration of PN in the first week. Infants will be matched on gestational age at birth (in bands: 30^{+0} to 30^{+6}, 31^{+0} to 31^{+6}, 32^{+0} to 32^{+6}), small for gestational age (treated as a dichotomous variable: <10th centile on the UK-WHO growth chart (33), or ≥10th centile) and propensity score. The propensity scores will be divided into propensity groups by the method of splitting proposed by Imbens and Rubin, with appropriate trimming of babies with unusually high or low scores (34). The propensity model will include maternal factors, infant factors at birth, infant factors occurring on the first day of birth (preceding the decision to administer PN) and organisational factors. A full list of background variables to be included in the propensity score can be found in eText 1.

We will calculate absolute risk differences and odds ratios for the pre-specified, dichotomous outcomes. All p values will be two-sided. We will use the Holm-Bonferroni method (35) when analysing secondary outcomes to avoid erroneous inferences due to the risk of false positives in multiple comparisons.

Subgroup analyses

As part of the descriptive survey we will compare PN use in infants of different gestational ages, different birth weights, in different geographical regions at neonatal network level (22), and compare how PN use has changed over the 5 year period.

For peer review only

To better replicate the research undertaken in adult and paediatric randomised controlled trials (8, 9) which defined early PN use as being before 48 hours we will undertake a sensitivity analysis comparing survival and all secondary outcomes in babies started on PN in the first 48 hours with those not receiving PN in the first seven postnatal days using propensity score matching. The sample size will be reduced but this analysis will provide further data for future research.

We will undertake a sensitivity analysis of the comparative study to explore the possibility that an unobserved variable explains any effect size seen to minimise the risk of findings due to confounding. We will construct a dichotomous variable to stack the odds against the superior treatment option and then compare this to the observed background variables to explore whether it is plausible that such an unobserved variable exists (36).

Patient and public involvement

This project was planned and designed with input from two parents of preterm infants who had experience of PN. This project will measure outcomes that we identified as most important to over 400 stakeholders with experience of neonatal care, including former patients and parents (27). Our study addresses three research priorities identified by the James Lind Alliance priority setting partnership for preterm birth (37), namely prevention of infection, lung damage and necrotising enterocolitis.

Ethics and dissemination

This study will only use de-identified data already held within the NNRD. The NNRD is UK Research Ethics Committee approved (REC Reference: 16/LO/1093) and Confidentiality Advisory Group approved (ECC 8-05(f/2010)) and all data is stored on NHS servers. Parents can opt out of their baby's data being held within the NNRD. Study specific REC approval and Health Research Authority and Health and Care Research Wales approval was obtained (18/NI/0214).

For peer review only

The results of this study will be presented at academic conferences and published in a peer-reviewed scientific journal. Bliss will aid dissemination in an appropriate form online and via social media.

Discussion

Uncertainty surrounds the use of PN in neonatal patients. Previous surveys of practice show that the use of PN in neonates is variable both nationally and internationally (17, 38, 39). It is not known how PN is used in neonatal units in England, Scotland and Wales nor how this has changed over time (40), and the gestational age at which the nutritional benefits of early PN outweigh the risks in moderately preterm babies is unknown. Clinical practice may vary due to the uncertainty surrounding the risks and benefits for individual babies; our study will give clinicians more evidence on which to base decisions about early use of PN.

The major limitation of any observational study is that the intervention is not randomly assigned and any differences in outcomes may be explained by confounding. We will address this issue using propensity score matching to generate two matched cohorts, analogous to the random allocation that would occur in a controlled trial. The NNRD contains comprehensive background data on both the infants and their mothers which will be included in the propensity score; these data have been demonstrated to be sufficiently robust for research purposes (23, 24). This means that we can ensure that any difference in outcomes seen is likely to be due to the exposure of interest rather than confounders. As propensity score matching only ensures that measured variables are balanced, we will undertake a sensitivity analysis to explore whether confounding due to an unmeasured variable is likely. Further strengths of this study are that data were entered by clinicians during routine care and so should not be subject to recall bias. The NNRD covers all

For peer review only

neonatal units in England, Wales and Scotland, hence "recruitment bias" due to incomplete population coverage is eliminated and findings will be generalisable to other populations.

In this work we will describe rates of PN use in neonatal units across England, Scotland and Wales for the first time. We will also describe the rates of important neonatal outcomes in moderately preterm neonates who receive early PN. This data will allow future randomised controlled trials to calculate expected PN exposure rates so that recruitment can be planned and provide outcome rates to allow accurate sample size calculation. These prospective studies would ensure that all other elements of care are equivalent, controlling for any possible confounding factors and providing conclusive evidence of a causal link between PN use and outcomes. Identifying whether moderately preterm neonates benefit from early PN will guide practice and also inform future research. At present there is uncertainty around optimal use of PN in preterm infants, this protocol describes a database study that is the first step in addressing this problem.

For peer review only

ABBREVIATIONS

COIN	Core Outcomes in Neonatology
NICU	Neonatal Intensive Care Unit
NDS	Neonatal Data Set
NHS	National Health Service
NNAP	National Neonatal Audit Project
NNRD	National Neonatal Research Database
REC	Research Ethics Committee
RCPCH	Royal College of Paediatrics and Child Health
SCBU	Special Care Baby Unit

For peer review only

17

DECLARATIONS

Consent for publication

Not applicable

Availability of data and material

Not applicable

Competing interests

JW has received support from Chiesi Pharmaceuticals to attend an educational conference and has received a research grant from Mason Medical Research Foundation.

SU has received funding from the National Institute of Health Research, the Department of Health and Prolacta Life Sciences. SU has been on the Advisory Board of Fresenius Kabi and received honoraria and travel expenses for speaking at study days organised by Fresenius Kabi. SU is a member of the National Institute for Health and Care Excellence Parenteral Nutrition Guideline Development Committee. NM is Director of the Neonatal Data Analysis Unit at Imperial College London. In the last five years NM has served on the Board of Trustees of the Royal College of Paediatrics and Child Health, David Harvey Trust, Medical Women's Federation and Medact; and is a member of the Nestle Scientific Advisory Board. NM has received research grants from the British Heart Foundation, Medical Research Council, National Institute of Health Research, Westminster Research Fund, Collaboration for Leadership in Applied Health and Care Northwest London, Healthcare Quality Improvement Partnership, Bliss, Prolacta Life Sciences, Chiesi, Shire and HCA International; travel and accommodation expenses from, Nutricia, Prolacta, Nestle and Chiesi; honoraria from Ferring Pharmaceuticals and Alexion Pharmaceuticals for contributions to expert advisory boards, and Chiesi for contributing to a lecture programme.

For peer review only

CG is part of an international team developing reporting guidance (a CONSORT extension) for clinical trials using cohorts and routinely collected health data. He has received support from Chiesi Pharmaceuticals to attend an educational conference; in the past 5 years he been investigator on received research grants from Medical Research Council, National Institute of Health Research, Canadian Institute of Health Research, Department of Health in England, Mason Medical Research Foundation, Westminster Medical School Research Trust and Chiesi Pharmaceuticals.

Funding

The funding for creating and maintaining the NNRD is from unrestricted funding awarded to NM. This includes costs involved in data transfer, storage, cleaning, merging, administration and regulatory approvals. The extraction of study data from the NNRD and analysis for this study is funded through a Mason Medical Research Fellowship awarded to JW. CG is funded by the United Kingdom Medical Research Council (MRC) through a Clinician Scientist Fellowship award. The MRC and Mason Medical Research Foundation were not involved in the design of the study, collection, analysis, and interpretation of data or in writing the manuscript.

Authors' contributions

JW, CG and NM conceived this project. JW, CG and NL planned the statistical analyses. The first draft of the manuscript was written by JW and revised by NM; CG, SU, NL and NM edited and reviewed the manuscript. It was approved by JW, CG, NL, SU and NM.

Acknowledgements

We wish to thank Angela Richard-Löndt and Laura Noakes, parents of preterm infants, for their support in developing this research project and BLISS for their input and support developing the dissemination plan.

For peer review only

19

REFERENCES

1. Fanaro S. Feeding intolerance in the preterm infant. Early Hum Dev. 2013;89:S13-S20.
2. Christensen RD, Henry E, Wiedmeier SE, Burnett J, Lambert DK. Identifying patients, on the first day of life, at high-risk of developing parenteral nutrition-associated liver disease. Journal of perinatology : official journal of the California Perinatal Association. 2007;27(5):284-90. Epub 2007/03/09.
3. Ksiazyk J, Swietlinski J, Brozek G, Musialik-Swietlinska E, Migdal M, Zejda JE. [Parenteral nutrition in neonatal units: a survey of current practices in Poland]. Medycyna wieku rozwojowego. 2008;12(4 Pt 1):899-904. Epub 2008/01/01. Zywienie pozajelitowe w oddzialach neonatologii w Polsce - analiza stosowanych praktyk.
4. British Association of Perinatal Medicine. The Provision of Parenteral Nutrition within Neonatal Services - A Framework for Practice. [Guideline] 2016 [updated April 2016; cited 2017 25/05/2017]; Available from: http://www.bapm.org/publications/documents/guidelines/Parenteral%20Nutrition%20April%202016.pdf.
5. Trivedi A, Sinn JKH. Early versus late administration of amino acids in preterm infants receiving parenteral nutrition. Cochrane Db Syst Rev. 2013(7).
6. Moyses HE, Johnson MJ, Leaf AA, Cornelius VR. Early parenteral nutrition and growth outcomes in preterm infants: a systematic review and meta-analysis. The American journal of clinical nutrition. 2013;97(4):816-26. Epub 2013/03/01.
7. Zingg W, Tomaske M, Martin M. Risk of parenteral nutrition in neonates--an overview. Nutrients. 2012;4(10):1490-503. Epub 2012/12/04.
8. Casaer MP, Mesotten D, Hermans G, Wouters PJ, Schetz M, Meyfroidt G, et al. Early versus late parenteral nutrition in critically ill adults. The New England journal of medicine. 2011;365(6):506-17. Epub 2011/07/01.
9. Fivez T, Kerklaan D, Mesotten D, Verbruggen S, Wouters PJ, Vanhorebeek I, et al. Early versus Late Parenteral Nutrition in Critically Ill Children. The New England journal of medicine. 2016;374(12):1111-22. Epub 2016/03/16.
10. van Puffelen E, Vanhorebeek I, Joosten KFM, Wouters PJ, Van den Berghe G, Verbruggen S. Early versus late parenteral nutrition in critically ill, term neonates: a preplanned secondary subgroup analysis of the PEPaNIC multicentre, randomised controlled trial. The Lancet Child & adolescent health. 2018;2(7):505-15. Epub 2018/09/01.
11. Heird WC, Gomez MR. Parenteral nutrition in low-birth-weight infants. Annu Rev Nutr. 1996;16:471-99.
12. Simmer K, Rao SC. Early introduction of lipids to parenterally-fed preterm infants. The Cochrane database of systematic reviews. 2005(2):CD005256. Epub 2005/04/23.
13. Uthaya S, Liu X, Babalis D, Dore CJ, Warwick J, Bell J, et al. Nutritional Evaluation and Optimisation in Neonates: a randomized, double-blind controlled trial of amino acid regimen and intravenous lipid composition in preterm parenteral nutrition. The American journal of clinical nutrition. 2016;103(6):1443-52. Epub 2016/04/22.
14. Tan MJ, Cooke RW. Improving head growth in very preterm infants - a randomised controlled trial I: neonatal outcomes. Arch Dis Child-Fetal. 2008;93(5):F337-F41.
15. Vlaardingerbroek H, Vermeulen MJ, Rook D, van den Akker CHP, Dorst K, Wattimena JL, et al. Safety and Efficacy of Early Parenteral Lipid and High-Dose Amino Acid Administration to Very Low Birth Weight Infants. Journal of Pediatrics. 2013;163(3):638-+.
16. Lanman JT, Guy LP, Dancis J. Retrolental fibroplasia and oxygen therapy. Journal of the American Medical Association. 1954;155(3):223-6. Epub 1954/05/15.
17. Lapillonne A, Kermorvant-Duchemin E. A systematic review of practice surveys on parenteral nutrition for preterm infants. The Journal of nutrition. 2013;143(12 Suppl):2061S-5S. Epub 2013/10/11.
18. Mason DG, Puntis JW, McCormick K, Smith N. Parenteral nutrition for neonates and children: a mixed bag. Arch Dis Child. 2011;96(3):209-10. Epub 2010/09/21.

For peer review only - http://bmjopen.bmj.com/site/about/guidelines.xhtml

19. Gale C, Morris I, NDAU NDAU. The UK National Neonatal Research Database: using neonatal data for research, quality improvement and more. Arch Dis Childhood-E. 2016;101(4):216-8.
20. Spencer A, Modi N. National neonatal data to support specialist care and improve infant outcomes. Archives of disease in childhood Fetal and neonatal edition. 2013;98(2):F175-80. Epub 2012/01/05.
21. NHS Digital. National Neonatal Data Set. 2017 [12/07/2017]; Available from: http://www.datadictionary.nhs.uk/data_dictionary/messages/clinical_data_sets/data_sets/national_neonatal_data_set/national_neonatal_data_set_-_episodic_and_daily_care_fr.asp?shownav=1.
22. Gale C, Santhakumaran S, Nagarajan S, Statnikov Y, Modi N, Unit NDA, et al. Impact of managed clinical networks on NHS specialist neonatal services in England: population based study. British Medical Journal. 2012;344.
23. Battersby C, Statnikov Y, Santhakumaran S, Gray D, Modi N, Costeloe K, et al. The United Kingdom National Neonatal Research Database: A validation study. PloS one. 2018;13(8).
24. Battersby C, Longford N, Mandalia S, Costeloe K, Modi N. Incidence and enteral feed antecedents of severe neonatal necrotising enterocolitis across neonatal networks in England, 2012-13: a whole-population surveillance study. The lancet Gastroenterology & hepatology. 2017;2(1):43-51. Epub 2017/04/14.
25. Fraser LK, Miller M, Hain R, Norman P, Aldridge J, McKinney PA, et al. Rising national prevalence of life-limiting conditions in children in England. Pediatrics. 2012;129(4):e923-9. Epub 2012/03/14.
26. Manktelow BN, Smith LK, Prunet C, Smith PW, Boby T, Hyman-Taylor P, et al. MBRRACE-UK Perinatal Surveillance Report: UK Perinatal Deaths for Births from January to December 2015. Leicester: The Infant Mortality and Morbidity Studies, Department of Health Sciences, University of Leicester, 2017.
27. Webbe J, Brunton G, Ali S, Duffy JM, Modi N, Gale C. Developing, implementing and disseminating a core outcome set for neonatal medicine. BMJ Paediatrics Open. 2017;1(1).
28. Royal College of Paediatrics and Child Health. National Neonatal Audit Programme A guide to the 2018 audit measures. Royal College of Paediatrics and Child Health, 2017.
29. Battersby C, Longford N, Patel M, Selby E, Ojha S, Dorling J, et al. Study protocol: optimising newborn nutrition during and after neonatal therapeutic hypothermia in the United Kingdom: observational study of routinely collected data using propensity matching. BMJ open. 2018;8(10):e026739. Epub 2018/10/26.
30. Papile LA, Burstein J, Burstein R, Koffler H. Incidence and Evolution of Subependymal and Intra-Ventricular Hemorrhage - Study of Infants with Birth Weights Less Than 1,500 Gm. Journal of Pediatrics. 1978;92(4):529-34.
31. Blencowe H, Cousens S, Chou D, Oestergaard M, Say L, Moller AB, et al. Born Too Soon: The global epidemiology of 15 million preterm births. Reprod Health. 2013;10.
32. World Health Organization. International Statistical Classification of Diseases and Related Health Problems 10th Revision. 2016.
33. Onis M, Onyango A, Borghi E, Siyam A, Pinol A, Garza C, et al. Enrolment and baseline characteristics in the WHO Multicentre Growth Reference Study. Acta Paediatrica. 2006;95:7-15.
34. Imbens GW, Rubin DB. Causal Inference for Statistics, Social, and Biomedical Sciences: An Introduction. Cambridge: Cambridge University Press; 2015.
35. Holm S. A Simple Sequentially Rejective Multiple Test Procedure. Scand J Stat. 1979;6(2):65-70.
36. Rosenbaum PR, Rubin DB. Assessing Sensitivity to an Unobserved Binary Covariate in an Observational Study with Binary Outcome. J Roy Stat Soc B Met. 1983;45(2):212-8.
37. Duley L, Uhm S, Oliver S. Top 15 UK research priorities for preterm birth. Lancet. 2014;383(9934):2041-2. Epub 2014/06/17.

For peer review only

38. Lapillonne A, Carnielli VP, Embleton ND, Mihatsch W. Quality of newborn care: adherence to guidelines for parenteral nutrition in preterm infants in four European countries. BMJ open. 2013;3(9):e003478. Epub 2013/09/21.
39. Ziegler EE, Thureen PJ, Carlson SJ. Aggressive nutrition of the very low birthweight infant. Clinics in perinatology. 2002;29(2):225-44. Epub 2002/08/10.
40. Koletzko B, Goulet O, Hunt J, Krohn K, Shamir R. 1. Guidelines on Paediatric Parenteral Nutrition of the European Society of Paediatric Gastroenterology, Hepatology and Nutrition (ESPGHAN) and the European Society for Clinical Nutrition and Metabolism (ESPEN), Supported by the European Society of Paediatric Research (ESPR). Journal of pediatric gastroenterology and nutrition. 2005;41 Suppl 2:S1-87. Epub 2005/10/29.

For peer review only

Online only supplemental material

eTable 1: Exclusion criteria for comparative study: list of major congenital gastrointestinal malformations
eTable 2: Exclusion criteria for comparative study: list of life-limiting conditions or conditions requiring surgery in the neonatal period
eText 1: Data fields for extraction from NNRD

For peer review only

eTable 1: Exclusion criteria for comparative study: list of major congenital gastrointestinal malformations

Clevermed code	ICD-10 code	Diagnosis
10741	Q39.0	Oesophageal atresia without distal fistula
16195	Q39.0	Atresia of oesophagus without fistula
10740	Q39.1	Oesophageal atresia with distal tracheo-oesophageal fistula
16196	Q39.1	Atresia of oesophagus with tracheo-oesophageal fistula (TOF)
16197	Q39.2	Congenital tracheo-oesophageal fistula without atresia (TOF)
10273	Q39.3	Congenital stenosis of the oesophagus
16198	Q39.3	Congenital stenosis and stricture of oesophagus
16199	Q39.4	Oesophageal web
10358	Q41.0	Duodenal atresia / stenosis / web (specify)
16212	Q41.0	Congenital absence, atresia and stenosis of duodenum
16213	Q41.0DA	Duodenal atresia / stenosis
10605	Q41.1	Jejunal atresia / stenosis (specify)
16214	Q41.1JA	Jejunal atresia / stenosis
10541	Q41.2	Ileal atresia / stenosis (specify)
16215	Q41.2	Congenital absence, atresia and stenosis of ileum
16216	Q41.2IA	Ileal atresia / stenosis
16217	Q41.X	Congenital absence, atresia and stenosis of small intestine
16218	Q42.0	Congenital absence, atresia and stenosis of rectum with fistula
10496	Q42.00	High anorectal anomaly with rectourethral fistula
10497	Q42.01	High anorectal anomaly with rectovesical fistula
10498	Q42.02	High anorectal anomaly with rectovulval fistula
10495	Q42.03	High anorectal anomaly with rectocutaneous fistula
10494	Q42.04	High anorectal anomaly with rectocloacal fistula
10493	Q42.08	High anorectal anomaly with fistula (specify)
10499	Q42.1	High anorectal anomaly without fistula
16219	Q42.1	Congenital absence, atresia and stenosis of rectum without fistula
16220	Q42.2	Congenital absence, atresia and stenosis of anus with fistula
10636	Q42.20	Low anorectal anomaly with anocutaneous fistula
10637	Q42.21	Low anorectal anomaly with anovestibular fistula
10638	Q42.28	Low anorectal anomaly with fistula (other specify)
10639	Q42.3	Low anorectal anomaly without fistula
16221	Q42.3	Congenital absence, atresia and stenosis of anus without fistula
10240	Q42.31	Congenital anal stenosis
16222	Q42.8	Congenital absence, atresia and stenosis of anus of other parts of large intestine
16223	Q429	Congenital absence, atresia and stenosis of anus of large intestine, part unspecified
16224	Q42X	Congenital absence, atresia and stenosis of large intestine

For peer review only

16235	Q43.7	Persistent cloaca

For peer review only

eTable 2: Exclusion criteria for comparative study: list of life-limiting conditions or conditions requiring surgery in the neonatal period

Clevermed code	ICD-10 code	Diagnosis
15890	Q00.0	Anencephaly
15891	Q00.1	Craniorachischisis
15892	Q00.2	Iniencephaly
15893	Q00.X	Anencephaly and similar malformations
15894	Q01.0	Frontal encephalocele
15895	Q01.1	Nasofrontal encephalocele
15896	Q01.2	Occipital encephalocele
15897	Q01.8	Encephalocele of other sites
15898	Q01.9	Encephalocele (unknown or unspecified cause)
15899	Q01.X	Encephalocele
15918	Q04.2	Holoprosencephaly
15926	Q05.0	Cervical spina bifida with hydrocephalus
15927	Q05.1	Thoracic spina bifida with hydrocephalus
15928	Q05.2	Lumbar spina bifida with hydrocephalus
15929	Q05.3	Sacral spina bifida with hydrocephalus
15930	Q05.4	(unknown or unspecified cause) spina bifida with hydrocephalus
15931	Q05.5	Cervical spina bifida without hydrocephalus
15932	Q05.6	Thoracic spina bifida without hydrocephalus
15933	Q05.7	Lumbar spina bifida without hydrocephalus
15934	Q05.8	Sacral spina bifida without hydrocephalus
15935	Q05.9	Spina bifida (unknown or unspecified cause)
10986	Q05.9a	Spina bifida
10704	Q05.9b	Myelomeningocele (specify site)
15936	Q05.X	Spina bifida
16024	Q20.0	Common arterial trunk (Truncus malformation)
10356	Q20.1	Double outlet right ventricle (DORV)
16025	Q20.1	Double outlet right ventricle (DORV)
16026	Q20.2	Double outlet left ventricle (DOLV)
11070	Q20.3	Transposition of the great vessels (TGA)
16027	Q20.3	Transposition great arteries (TGA)
16028	Q20.4	Double inlet ventricle (DILV)
16029	Q20.5	Discordant atrioventricular connection
16030	Q20.6	Isomerism of atrial appendages
16031	Q20.8	Other cong malforms of cardiac chambers and connections
16032	Q20.9	Cong malforms of cardiac chambers and connections unspec
16033	Q20.X	Congenital malformations of cardiac chambers and connections
16035	Q20.91	Atrium single

For peer review only

16036	Q20.92	Ventricle single
10097	Q21.2	Atrio-ventricular septal defect (AVSD)
16039	Q21.2	Atrioventricular septal defect (AVSD)
11043	Q21.3	Tetralogy of Fallot
16040	Q21.3	Tetralogy of Fallot
16045	Q22.0	Pulmonary valve atresia
16046	Q22.1	Congenital pulmonary valve stenosis
16047	Q22.2	Congenital pulmonary valve insufficiency
16048	Q22.3	Other congenital malformations of pulmonary valve
16049	Q22.4	Congenital tricuspid atresia / stenosis
16050	Q22.5	Ebstein's anomaly
16051	Q22.6	Hypoplastic right heart syndrome
16052	Q22.8	Other congenital malformations of tricuspid valve
16053	Q22.9	Congenital malformation of tricuspid valve (unknown or unspecified cause)
16054	Q22.X	Congenital malformations of pulmonary and tricuspid valves
16055	Q23.0	Congenital stenosis of aortic valve (AS)
16056	Q23.1	Congenital insufficiency of aortic valve
16057	Q23.2	Congenital mitral stenosis (MS)
16058	Q23.3	Mitral atresia
16059	Q23.4	Hypoplastic left heart syndrome (HLH)
16060	Q23.8	Other congenital malformations of aortic and mitral valves
16061	Q23.9	Congenital malformation of aortic and mitral valves unspec
16062	Q23.X	Congenital malformations of aortic and mitral valves
16079	Q25.1	Coarctation of aorta
10227	Q25.19	Coarctation of the aorta
16080	Q25.2	Hypoplasia of aortic arch
16081	Q25.3	Stenosis of aorta (AS)
16082	Q25.4	Malformation of aorta
16083	Q25.5	Atresia of pulmonary artery
16084	Q25.6	Stenosis of pulmonary artery (PS)
16086	Q25.8	Other congenital malformations of great arteries
16087	Q25.8	Transposition of the great vessels (TGA)
11057	Q26.2	Total anomylous pulmonary venous drainage (TAPVD)
16092	Q26.2	Total anomalous pulmonary venous connection (TAPVD)
16154	Q33.6	Hypoplasia and dysplasia of lung
16241	Q44.2	Atresia of bile ducts
10123	Q60.1	Bilateral renal agenesis
16318	Q60.1B	Renal agenesis, bilateral
16324	Q60.6	Potter's syndrome
16327	Q61.1	Polycystic kidney, infantile type
10100	Q61.1a	Autosomal recessive polycystic kidney - infantile

For peer review only

10367	Q64.1	Ectopia vesicae
16356	Q64.1	Exstrophy of urinary bladder
10854	Q64.2	Posterior urethral valves (PUV)
16357	Q64.2	Congenital posterior urethral valves (PUV)
16360	Q64.5	Congenital absence of bladder and urethra
10008	Q64.5a	Absence of bladder
10236	Q64.5b	Congenital absence of urethra
16475	Q77.1	Thanatophoric short stature
10246	Q79.0	Congenital diaphragmatic hernia
10490	Q79.0	Hernia into the cord
16495	Q79.0	Congenital diaphragmatic hernia
16496	Q79.1A	Aplasia of diaphragm
16497	Q79.1E	Eventration of diaphragm
16498	Q79.2	Exomphalos
10395	Q79.2	Exomphalos
16499	Q79.3	Gastroschisis
16589	Q90.0	Trisomy 21, meiotic nondisjunction
16590	Q90.1	Trisomy 21, mosaicism (mitotic nondisjunction)
16591	Q90.2	Trisomy 21, translocation
16592	Q90.9	Down's syndrome (unknown or unspecified cause)
16593	Q90.X	Down's syndrome
16594	Q91.0	Trisomy 18, meiotic nondisjunction
16595	Q91.1	Trisomy 18, mosaicism (mitotic nondisjunction)
16596	Q91.2	Trisomy 18, translocation
16597	Q91.3	Edwards' syndrome (unknown or unspecified cause)
16598	Q91.4	Trisomy 13, meiotic nondisjunction
16599	Q91.5	Trisomy 13, mosaicism (mitotic nondisjunction)
16600	Q91.6	Trisomy 13, translocation
16601	Q91.7	Patau's syndrome (unknown or unspecified cause)
16602	Q91.X	Edwards' syndrome and Patau's syndrome

For peer review only

eText 1: Data fields for extraction from NNRD

Treatment	
**Variable**	**Data items**
Parenteral nutrition	**PN group** defined as<br>Any of the following items entered in the 'Daily Care Fluids' and 'Feeding' during first 7 days:<br>• Y entry for PARENTERAL NUTRITION RECEIVED INDICATOR<br>Or<br>The following drug code entered in the Daily care medication during first 7 days:<br>• 1010238 Total parenteral nutrition<br><br>**No PN group** defined as<br>All other babies

For peer review only

Background variables for matching	
**Variable**	**Data items**
Gestational age at birth	**30^{+0} to 30^{+6} group** defined as<br>Any of the following items entered in the GESTATION LENGTH (AT DELIVERY):<br>• 30^{+0}, 30^{+1}, 30^{+2}, 30^{+3}, 30^{+4}, 30^{+5}, 30^{+6}<br><br>**31^{+0} to 31^{+6} group** defined as<br>Any of the following items entered in the GESTATION LENGTH (AT DELIVERY):<br>• 31^{+0}, 31^{+1}, 31^{+2}, 31^{+3}, 31^{+4}, 31^{+5}, 31^{+6}<br><br>**32^{+0} to 32^{+6} group** defined as<br>Any of the following items entered in the GESTATION LENGTH (AT DELIVERY):<br>• 32^{+0}, 32^{+1}, 32^{+2}, 32^{+3}, 32^{+4}, 32^{+5}, 32^{+6}
Small for gestational age	**Small for gestational age group** defined as<br>Any result entered in the BIRTH WEIGHT which is below the 10th centile on the UK-WHO growth chart<br><br>**Appropriate for gestational age group** defined as<br>All other babies

Background variables for propensity score matching	
**Variable**	**Data items**
Sex	Data will be extracted from PERSON PHENOTYPIC SEX<br>• Categorical: 1 Male / 2 Female / 9 Indeterminate (unable to be classified as either male or female)
Multiplicity	Data will be extracted from NUMBER OF FETUSES (NOTED DURING PREGNANCY EPISODE); this excludes fetus papyraceous and fetuses reabsorbed in utero and not delivered.
Year of birth	Data will be extracted from DATE TIME OF BIRTH<br>• Continuous in one year bands
Maternal age	Data will be extracted from YEAR OF BIRTH (MOTHER)<br>• Continuous variable
Maternal diabetes	Data will be extracted from MATERNITY COMPLICATING MEDICAL DIAGNOSIS TYPE (NATIONAL NEONATAL DATA SET)<br>• Dichotomous: 08 Y/N
Maternal gestational	Data will be extracted from MATERNITY OBSTETRIC DIAGNOSIS

diabetes	TYPE (CURRENT PREGNANCY)<br>• Dichotomous: 06 Gestational diabetes mellitus Y/N
Maternal severe pre-eclampsia requiring pre-term birth	Data will be extracted from MATERNITY OBSTETRIC DIAGNOSIS TYPE (CURRENT PREGNANCY)<br>• Dichotomous: 01 Severe pre-eclampsia requiring pre-term birth Y/N
Maternal severe pre-eclampsia	Data will be extracted from MATERNITY OBSTETRIC DIAGNOSIS TYPE (CURRENT PREGNANCY)<br>• Dichotomous: 20 Severe pre-eclampsia Y/N
Maternal gestational hypertension	Data will be extracted from MATERNITY OBSTETRIC DIAGNOSIS TYPE (CURRENT PREGNANCY)<br>• Dichotomous: 07 Gestational hypertension Y/N
Maternal prolonged rupture of membranes	Data will be extracted from NUMBER OF MINUTES (BIRTH TO EVENT)<br>• Continuous variable
Maternal suspected chorioamnionitis	Data will be extracted from SIGNIFICANT MATERNAL PYREXIA IN LABOUR INDICATOR or INTRAPARTUM ANTIBIOTICS GIVEN INDICATOR<br>• Dichotomous: Suspected chorioamnionitis defined as Y in either field<br>• Dichotomous: No suspected chorioamnionitis defined as N in both fields
Maternal receipt of antenatal steroids	Data will be extracted from STEROIDS GIVEN DURING PREGNANCY TO MATURE FETAL LUNGS INDICATOR (Y/N) and ANTENATAL STEROID COURSE COMPLETION STATUS<br>• Categorical: Complete course defined as Y and 1 Complete course<br>• Categorical: Incomplete course defined as Y and 2 Incomplete course<br>• Categorical: No steroids defined as N
Maternal receipt of antenatal magnesium sulphate	Data will be extracted from MOTHER RECEIVED MAGNESIUM SULPHATE IN 24 HOURS PRIOR TO DELIVERY<br>• Dichotomous: Y/N
Infant Apgar score at 5 minutes	Data will be extracted from APGAR SCORE (5 MINUTES)<br>• Categorical: 0-10
Infant: chest compressions administered	Data will be extracted from NEONATAL RESUSCITATION METHODS (NATIONAL NEONATAL DATA SET)<br>• Dichotomous: 16 Cardiac massage (Y/N)
Infant: Emergency resuscitation drugs administered	Data will be extracted from NEONATAL RESUSCITATION METHODS (NATIONAL NEONATAL DATA SET)<br>• Dichotomous: 17 Adrenaline or 88 Any other drug (Y/N)
Infant: Intubated at resuscitation	Data will be extracted from NEONATAL RESUSCITATION METHODS (NATIONAL NEONATAL DATA SET)<br>• Dichotomous: 15 Intubation (Y/N)
Infant: Surfactant administered	Data will be extracted from SURFACTANT GIVEN INDICATOR (DURING RESUSCITATION)<br>• Dichotomous: Y/N
Infant: Umbilical cord pH	Data will be extracted from UMBILICAL CORD BLOOD PH LEVEL (ARTERIAL)<br>• Continuous: Arterial cord pH (6.00-8.00)<br>Or if unavailable use:<br>UMBILICAL CORD BLOOD PH LEVEL (VENOUS)<br>• Continuous: Venous cord pH (6.00-8.00)
Infant: Admission temperature	Data will be extracted from TEMPERATURE (ON ADMISSION TO NEONATAL CRITICAL CARE)<br>• Continuous
Infant: Admission mean blood pressure	Data will be extracted from MEAN ARTERIAL BLOOD PRESSURE (ON ADMISSION TO NEONATAL CRITICAL CARE)

For peer review only

	• Continuous: 10-150
Infant: Admission blood glucose	Data will be extracted from BLOOD GLUCOSE CONCENTRATION (ON ADMISSION TO NEONATAL CRITICAL CARE)<br>• Continuous: 0.0-50.0
Infant: Admission heart rate	Data extracted from HEART RATE (ON ADMISSION TO NEONATAL CRITICAL CARE)<br>• Continuous: 50-350
Infant: Admission respiratory rate	Data extracted from RESPIRATORY RATE (ON ADMISSION TO NEONATAL CRITICAL CARE)<br>• Continuous: 10-200
Infant: Admission oxygen saturation	Data extracted from OXYGEN SATURATION (ON ADMISSION TO NEONATAL CRITICAL CARE)<br>• Continuous: 10-100
Infant: Surfactant administered on first day	Data extracted from SURFACTANT GIVEN INDICATOR (ON NEONATAL CRITICAL CARE DAILY CARE DATE)<br>• Continuous: Y/N
Infant: Mechanical ventilation on first day	Data extracted from RESPIRATORY SUPPORT DEVICE TYPE (NATIONAL NEONATAL DATA SET) for first day<br>• Mechanical ventilation defined as 1 Endotracheal tube<br>• No ventilation defined as any other answer
Infant: Inotropes administered on first day	Data extracted from INOTROPE INFUSION RECEIVED INDICATOR for first day<br>• Dichotomous: Y/N<br>Or DAILY CARE MEDICATION on day 1 only<br>• 500098 Dopamine<br>• 500096 Dobutamine<br>• 500056 Adrenaline<br>• 500210 Noradrenaline<br>• 500116 Hydrocortisone<br>• 1010173 Milrinone
Infant: Sepsis suspected on first day	Data extracted from DAILY CARE INFECTIONS SEPSIS SUSPECTED INDICATOR for first day<br>• Y/N
Infant: Transfer on first day	Data extracted from Admission Details SITE CODE (OF ADMITTING NEONATAL UNIT) or ORGANISATION CODE (OF ADMITTING NEONATAL UNIT) is different from Baby Demographics SITE CODE (OF ACTUAL PLACE OF DELIVERY) or ORGANISATION CODE (OF ACTUAL PLACE OF DELIVERY)<br>And Baby Demographics EPISODE NUMBER is >1
Level of initial neonatal unit	Data extracted from SITE CODE (OF ACTUAL PLACE OF DELIVERY)
Neonatal network	Data extracted from SITE CODE (OF ACTUAL PLACE OF DELIVERY)
Enteral feeding	Data extracted from DAILY CARE FLUIDS AND FEEDING ENTERAL FEED TYPE GIVEN on day 1 and 2<br>• Categorical: Only maternal milk feeding defined as any of 1 Breastfeeding, 2 Mothers fresh expressed breast milk, 3 Mothers frozen expressed breast milk on either day with no other code.<br>• Categorical: Only donor milk feeding defined as 4 Donor expressed breast milk on either day with no other code.<br>• Categorical: Only formula defined as only 6 Formula milk on either day with no other code.<br>• Categorical: Not feeding defined as 9 - Not applicable (nil by mouth) on both days with no other code.<br>• Categorical: Mixed feeding as any combination of codes not consistent with the above categories.

For peer review only

Outcomes	
**Variable**	**Data items**
Survival	Data extracted from DISCHARGE DESTINATION FROM NEONATAL CRITICAL CARE<br>• Survival defined as any of 1, 2, 4, 5, 6<br>• Died defined as code 3, Died
Late onset sepsis	NNAP definition<br>Defined from Infection Cultures (Episodic) recorded after day 3<br>• Pure growth of pathogen from blood OR Pure growth of pathogen from CSF OR Either a pure growth of a skin commensal or a mixed growth with ≥3 clinical signs at the time of blood sampling
Necrotising enterocolitis	NNAP definition<br>Defined from DISCHARGE DETAILS based on WAS NEC DIAGNOSED THIS ADMISSION answer Y<br>With at least one clinical feature from:<br>• Bilious gastric aspirate or emesis/Abdominal distension/Occult or gross blood in stool (no fissure)<br>And at least one radiographic feature from:<br>• Pneumatosis/Hepato-biliary gas/Pneumoperitoneum<br><br>Where NNAP definition not recorded (e.g. prior to 2016):<br>Defined from Daily Care Gastrointestinal at any point during neonatal unit stay:<br>• Any entry (1 or 2) for TREATMENT TYPE FOR NECROTISING ENTEROCOLITIS<br>Or: From diagnostic codes •<br>• 1010683 Necrotising enterocolitis – suspected<br>• 10708 Necrotising enterocolitis – Perforated<br>• 15809 Necrotizing enterocolitis<br><br>AND<br><br>5 or more days nil by mouth defined from the Daily Care Fluids and Feeding for a continuous period of 5 days<br>• No under ENTERAL FEED TYPE GIVEN<br>• No entry under FORMULA MILK OR MILK FORTIFIER TYPE<br>• No value OR 0 for TOTAL VOLUME OF MILK RECEIVED<br>• No entry under ENTERAL FEEDING METHOD<br>While also receiving 5 or more days of antibiotics over the same 5 days as the baby was nil by mouth<br>Defined as 5 consecutive days of any of the following Daily care medication<br>• 1010155 Benzyl Penicillin<br>• 1010158 Augmentin<br>• 1010179 Flucloxicillin<br>• 500012 Flucloxacillin<br>• 500016 Gentamicin<br>• 500072 Co-amoxiclav<br>• 500086 Co-amoxiclav<br>• 500084 Ciprofloxacin<br>• 500029 Netilmicin<br>• 500002 Amikacin<br>• 500211 Tazocin<br>• 500023 Metronidazole<br>• 500040 Vancomycin<br>• 500007 Cefotaxime<br>• 500004 Ampicillin

For peer review only

For peer review only - http://bmjopen.bmj.com/site/about/guidelines.xhtml

	• 500009 Cefuroxime<br>• 500008 Ceftazidime<br>• 500175 Ceftriaxone<br>• 500032 Piperacillin<br>• 500206 Oflacillin<br>• 500005 Azlocillin<br>• 1010171 Linezolid<br>• 1010271 Cefalexin<br>• 1010139 Amoxicillin<br>• 500070 Amoxicillin<br>• 500128 Meropenem<br>• 500118 Imepenem<br>• 500145 Imipenem
Brain injury on imaging	Data extracted from CRANIAL ULTRASOUND SCANS (EPISODIC)<br>Brain injury defined as:<br>• INTRAVENTRICULAR HAEMORRHAGE GRADE (RIGHT SIDE) or INTRAVENTRICULAR HAEMORRHAGE GRADE (LEFT SIDE) code 3 or 4 (Grade 3/4 intraventricular haemorrhage)<br>Or:<br>• CYSTIC PERIVENTRICULAR LEUKOMALACIA OBSERVED DURING CRANIAL ULTRASOUND SCAN INDICATOR answer Y
Retinopathy of prematurity	Data extracted from RETINOPATHY OF PREMATURITY SCREENING (EPISODIC)<br>Retinopathy of prematurity defined as:<br>• RETINOPATHY OF PREMATURITY STAGE (LEFT EYE) or RETINOPATHY OF PREMATURITY STAGE (RIGHT EYE) any code except 0 (None seen)
Bronchopulmonary dysplasia	NNAP definition<br>Significant bronchopulmonary dysplasia defined as:<br>• DAILY SUMMARY at 36^{+0} receiving any respiratory support
Need for surgical procedures	Data extracted from PROCEDURE (OPCS ON NEONATAL CRITICAL CARE DAILY CARE DATE)<br>Surgery defined as any of the following codes:<br>• 100033 Surgery for meconium ileus (von)<br>• 100076 Skin or soft tissue surgery requiring general or spinal anesthesia (Description Required<br>• 11222 Closure of small intestine/ileal perforation<br>• 11501 Laparoscopy<br>• 11904 Colostomy<br>• 11905 Ileostomy<br>• 1010826 Major surgery<br><br>Or:<br>• Daily item ANY MAJOR SURGERY TODAY answer Y
Seizures	Seizure defined as:SEIZURE OCCURRED INDICATOR<br>• Y<br>Or: DIAGNOSIS (ICD RECORDED ON DISCHARGE FROM NEONATAL CRITICAL CARE) with code<br>• 10957 Seizures<br>• 15192 Seizure disorder<br>• 15194 Seizure disorder (cause unknown)<br>• 15195 Status epilepticus<br>• 15848 Seizures
Weight	Data extracted from Daily Care General Information PERSON WEIGHT IN GRAMS from final day
Head circumference	Data extracted from Daily Care General Information HEAD

For peer review only

	CIRCUMFERENCE IN CENTIMETRES from final day

Long Term Outcomes	
**Variable**	**Data items**
Blindness	Defined as an answer of Yes to the question “Does this child have a visual impairment?” on the NNAP form
Deafness	Defined as an answer of Yes to the question “Does this child have a hearing impairment?” on the NNAP form
Ability to walk	Defined as an answer of Yes to the question “Is this child unable to walk without assistance?” on the NNAP form

For peer review only
